# Architecture-Based Programming of Polymeric Micelles to Undergo Sequential Mesophase Transitions

**DOI:** 10.1021/acsmacrolett.3c00153

**Published:** 2023-06-05

**Authors:** Parul Rathee, Nicole Edelstein-Pardo, Francesca Netti, Lihi Adler-Abramovich, Amit Sitt, Roey J. Amir

**Affiliations:** † School of Chemistry, Faculty of Exact Sciences, Tel-Aviv University, Tel-Aviv 6997801, Israel; ‡ The Center for Physics and Chemistry of Living Systems, Tel-Aviv University, Tel Aviv 6997801, Israel; § The Center for Nanoscience and Nanotechnology, Tel-Aviv University, Tel Aviv 6997801, Israel; ∥ Department of Oral Biology, The Goldschleger School of Dental Medicine, Faculty of Medicine, Tel Aviv University, Tel Aviv 6997801, Israel; ⊥ ADAMA Center for Novel Delivery Systems in Crop Protection, Tel-Aviv University, Tel Aviv 6997801, Israel

## Abstract

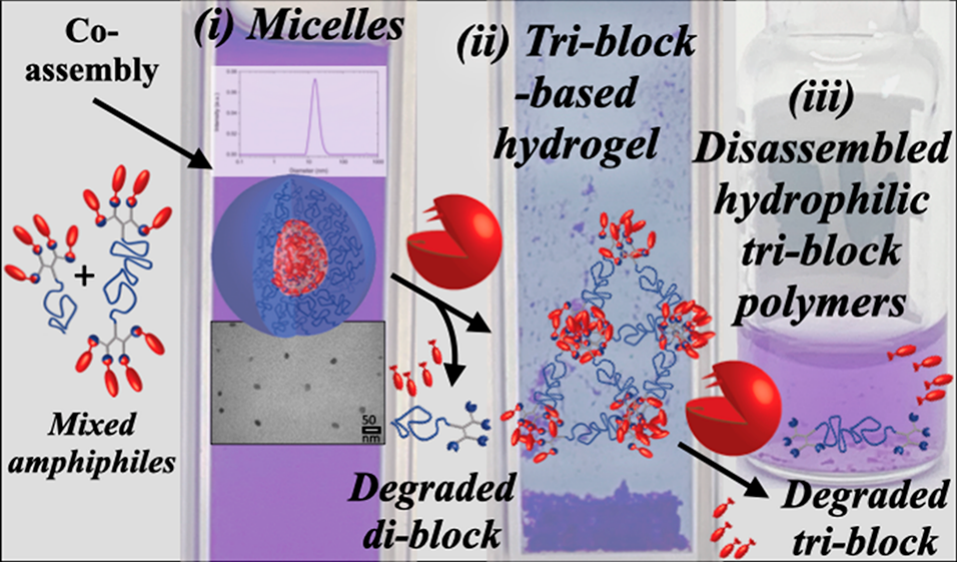

Di- and triblock amphiphiles can form different mesophases ranging from micelles to hydrogels depending on their chemical structures, hydrophilic to hydrophobic ratios, and their ratio in the mixture. In addition, their different architectures dictate their exchange rate between the assembled and unimer states and consequently affect their responsiveness toward enzymatic degradation. Here we report the utilization of the different reactivities of di- and triblock amphiphiles, having exactly the same hydrophilic to lipophilic balance, toward enzymatic degradation as a tool for programming formulations to undergo sequential enzymatically induced transitions from (i) micelles to (ii) hydrogel and finally to (iii) dissolved polymers. We show that the rate of transition between the mesophases can be programmed by changing the ratio of the amphiphiles in the formulation, and that the hydrogels can maintain encapsulated cargo, which was loaded into the micelles. The reported results demonstrate the ability of molecular architecture to serve as a tool for programming smart formulations to adopt different structures and functions.

The ability of supramolecular assemblies in Nature to alter their structure and function in response to multiple cues in their environment has inspired the development of stimuli-responsive polymeric amphiphiles and assemblies.^1−4^ Among the different types of stimuli, the overexpression of various disease-associated enzymes makes them highly promising for biomedical applications.^5−8^ While enzymatically triggered disassembly can be applied toward selective drug release at the site of disease and clearance of the delivery system,^7^ enzymatically induced self-assembly^9−11^ or aggregation (EISA),^12,13^ can be applied toward selective accumulation of polymeric-based depots for prolonged drug release at the target site.^14^ The programming of the type and sequence of these mesophase transitions is based on the incorporation of enzyme-responsive components in the amphiphiles. These can include enzymatically modifiable groups such as tyrosine and serine residues, which may undergo phosphorylation or dephosphorylation,^15−17^ and enzymatic cleavage sites.^18−24^ The enzymatic modification of the amphiphiles, which occurs at the molecular level, switches their polarity, which consequently translates to a transition into a different mesophase in the macroscale. Most enzyme-responsive systems contain a single type of responsive unit^25,26^ and hence can be programmed for a single transition between two mesophases, such as from micelles into hydrogels^27,28^ or aggregates^29^ or from soluble polymers to polymeric assemblies.^30,31^

The programming of materials to undergo multiple transitions between several mesophases can be extremely valuable for various applications such as controlled drug delivery systems.^32−36^ To allow both their rapid circulation in the body and selective accumulation at the site of disease, such carriers should potentially switch from stable nanostructures into soft polymeric hydrogels.^37^ After the release of their cargo, the aggregated polymers should change their mesophase again and transform into soluble polymers that can be readily cleared from the body.^38^ The design of materials that can undergo multiple sequential mesophase transitions requires the incorporation of different types of responsive units into the polymeric system.^39^ This has been demonstrated by the inspiring work of the Gianneschi group, which reported the ability to program polymers to transition between three mesophases by including two enzyme-responsive sites in each amphiphile so it can respond to two different enzymatic stimuli.^40^

Here we show that a single type of responsive unit can be used to achieve sequential multistep mesophase transitions by incorporating it into two amphiphiles with different architectures (Figure 1a). Over the past decade, we studied the ability to tune the stability of enzyme-responsive micelles by adjusting the molecular weight^41^ and hydrophilic to lipophilic balance (HLB)^42,43^ of PEG-dendron diblock amphiphiles (DBA). Recently, we expanded their architecture into triblock (hydrophobic–hydrophilic–hydrophobic) amphiphiles (TBA) composed of dendrons as hydrophobic side blocks and used them to prepare microparticles by electrospinning.^44^ When placing the microparticles in water, they swelled into hydrogel particles, which stayed stable for days to months, depending on the degree of hydrophobicity. Similarly, a hydrogel was formed when directly introducing the TBA to water regardless of whether thin-film hydration or solvent exchange was applied (Figure S25).

**Figure 1:**
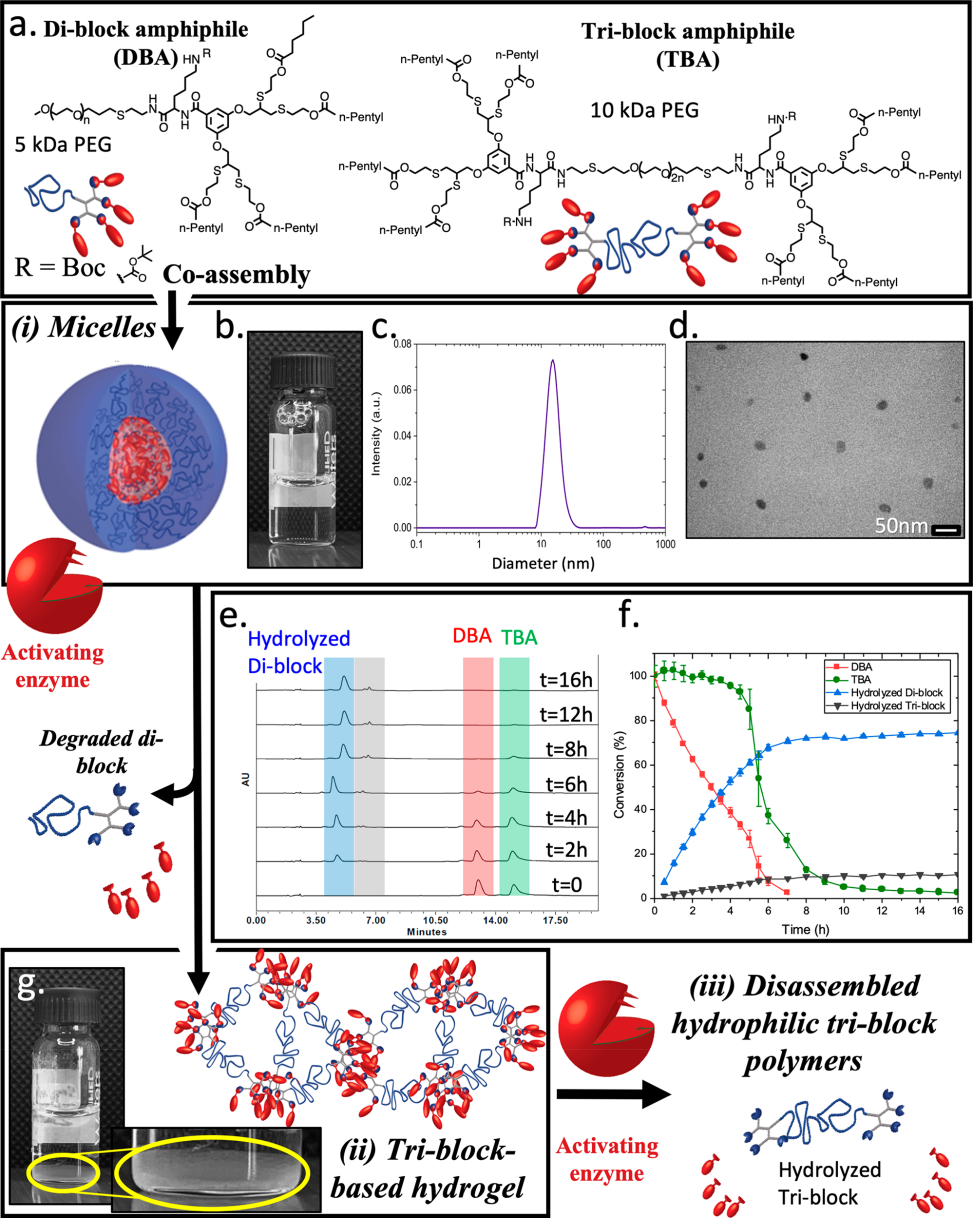
Schematic illustration of programming sequential mesophase transitions: (a) DBA and TBA amphiphiles are co-assembled into (i) mixed micelles ((b) photo, (c) DLS data, and (d) TEM image of the micellar solution). Faster enzymatic cleavage of the hydrophobic end-groups (in red) of DBA ((e) overlay of HPLC chromatograms and (f) kinetic data) leads to an increase in TBA concentration, causing its aggregation into (ii) a hydrogel ((g) photo of the hydrogel). Finally, slow enzymatic degradation of the TBA results in additional mesophase transition into (iii) soluble polymers.

Aiming to decrease the stability of the hydrogels so a micellar mesophase can be stabilized,^45^ we mixed TBA with DBA having identical dendron and consequently HLB by using a PEG chain with exactly half the molecular weight for the DBA (Figure 1a). DBA and TBA were mixed at a 1:1 ratio in organic solvent to obtain maximal blending, followed by evaporation of the solvent to yield a thin film. Hydration of the film using PBS buffer^46,47^ resulted in the desired formation of micelles with sizes of around 20 nm as indicated by DLS and TEM (Figures 1b–d).

Encouraged by the formation of mixed micelles, we studied their activation with porcine liver esterase (PLE) as a model enzyme, which can cleave the hexanoate end-groups.^48^ Micellar solutions (1:1 DBA:TBA) were incubated with the activating enzyme, and HPLC was used to directly monitor the molecular composition of the solution (Figure 1e). Initially, two peaks corresponding to the two types of amphiphiles with the expected 1:1 ratio were observed. Interestingly, we observed high selectivity of the enzyme toward degradation of the DBA, which reached nearly 70% after 5 h, while the TBA remained nearly intact (∼10% degradation). Next, we observed a sudden drop of around 50% in the area of the TBA peak, followed by further decrease in the concentrations of both types of amphiphiles (Figure 1f). Remarkably, the chromatograms did not indicate an extensive simultaneous formation of cleaved triblock, implying that the disappearance of the TBA is not a result of its enzymatic degradation. When looking at the HPLC vial (Figure 1g), the formation of hydrogel was clearly observed, explaining the rapid decrease in TBA concentration. As a control, we also monitored the enzymatic degradation of micelles made from only DBA, which showed faster degradation (Figures S18 and S19), providing further support for the formation of mixed micelles when the two types of amphiphiles are mixed. The results, which are in good agreement with our previous report of splittable TBA^49^ and gemini amphiphiles,^50^ indicate that the DBA could rapidly exchange between the micellar and unimer states, thus being highly accessible to the activating enzyme.^51^ On the other hand, the higher molecular weight and architecture of the TBA, as was also described by Lodge and Bates for other TBA-based assemblies,^52,53^ made the TBA exchange significantly slower and hence nearly unaffected by the degrading enzyme during the initial micellar state. Once the concentration of DBA decreased below a certain threshold (∼0.3 DBA to 1 TBA), it could no longer stabilize the micellar mesophase, and the increase in the relative concentration of TBA triggered the transition into a hydrogel. To analyze the composition of the formed hydrogels, they were filtered and dissolved in acetonitrile, which is a good solvent for both blocks. The analysis (Figure S23) showed the expected presence of the TBA and a low amount (∼10%) of the unhydrolyzed DBA, thus explaining the further drop in DBA concentration after gelation.

To further demonstrate the co-assembly, we labeled the amphiphiles with fluorescent markers that can undergo Förster resonance energy transfer (FRET).^54^ DBA were labeled with Cy5, as FRET acceptor, and TBA were labeled with Cy3, as a donor. Fluorescence spectra of the mixed labeled micelles (10% labeling) were collected by exciting the sample at 512 nm (Cy3 excitation) and measuring the emission of Cy3 (575 nm) and Cy5 (700 nm). The results showed a very strong FRET signal at 700 nm, providing vital evidence for the co-assembly (Figure 2a). Next, we followed both the overall emission intensity of both dyes and ratio of the Cy5 and Cy3 emissions after adding the activating enzyme to the labeled co-assembled micelles (Figure 2b). The total emission rapidly decreased by nearly 50% in the first 5 h and then kept decreasing but with a much milder rate. On the other hand, the Cy5:Cy3 ratio reduced by around 20% in the first 4 h, followed by a nearly 20-fold decrease from 4 to 6 h. These results correlate well with the HPLC analysis of the nonlabeled amphiphiles that showed an initial stage of degradation of the DBA (in the first 4–5 h), followed by the transition of the remaining TBA into hydrogel in the next stage (after 5 h). As the Cy5-labeled DBA become more hydrophilic after enzymatic degradation of their hydrophobic end-groups, they diffuse away from the micelles and become too far from the effective FRET distance from the Cy3-labeled triblocks that remain assembled, causing a decrease in both the total fluorescence and ratio. In addition, the aggregation of the remaining TBA into hydrogels and their precipitates out of solution at around 4 h also causes the decrease in overall emission as the effective concentrations of the labeling dyes in the solution substantially decrease. In addition, a photo of the sample shows that, initially, the micellar solution has a clear purple color due to the presence of both dyes. Photos of this sample after the addition of PLE showed the expected formation of a purple hydrogel, due to the presence of both dyes in the aggregated hydrogel, while the solution became bluer due to the change in the ratio of Cy5 and Cy3 amphiphiles, in comparison with the initial conditions (Figure 2b). The stability of the mixed micelles in the absence of the enzyme was confirmed by HPLC, DLS, and fluorescence measurements (Figures S15, S17a, and S24).

**Figure 2:**
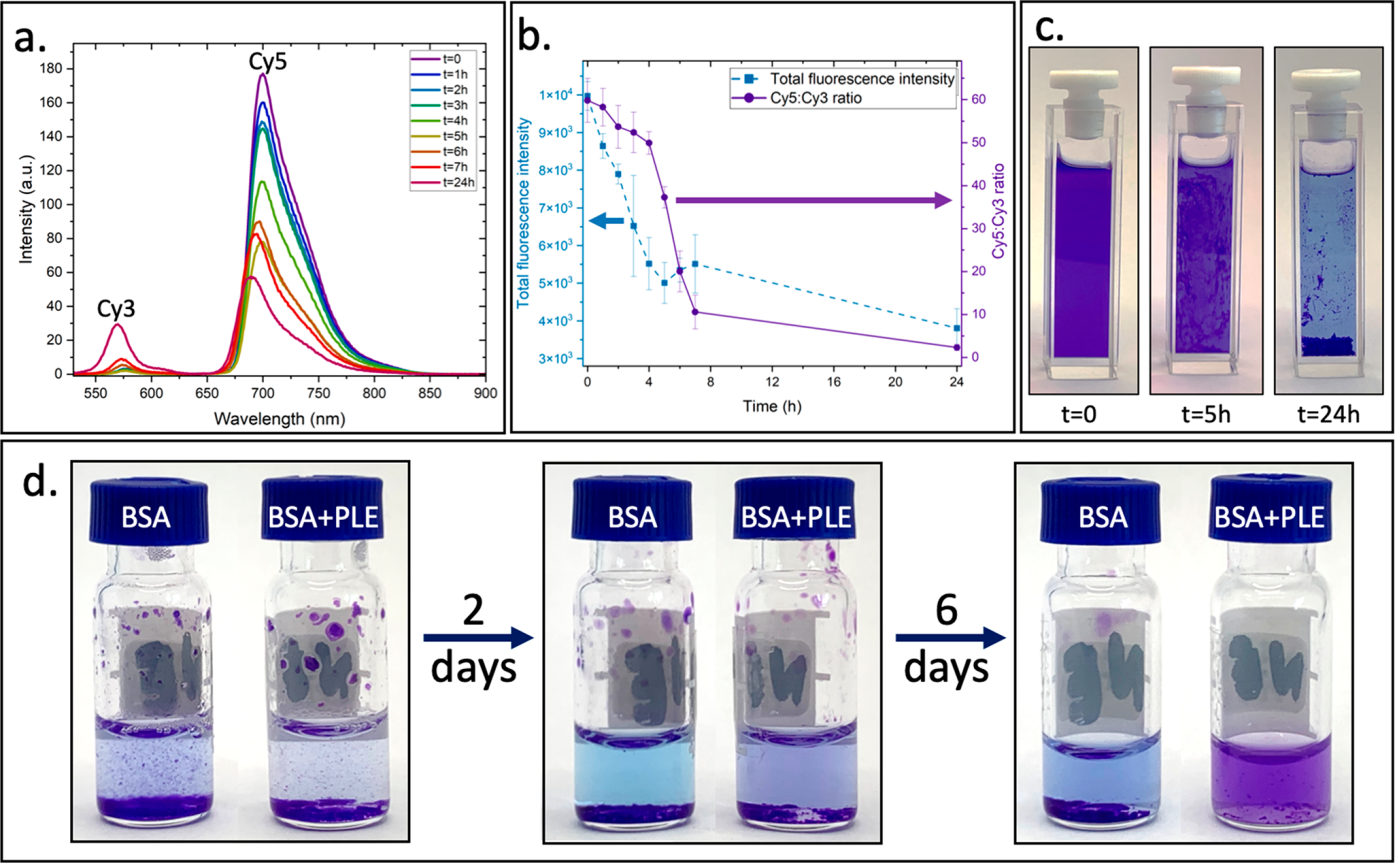
(a) Fluorescence spectra of micelles containing both dyes. (b) Overall intensity and Cy5:Cy3 emissions ratio as a function of time after the addition of PLE. (c) Photos of the solutions at different time points. (d) Photos of the vials containing hydrogel with BSA or BSA and PLE over 8 days, indicating the hydrogel transform into hydrolyzed polymers in the presence of PLE.

After demonstrating the transition from micelles to hydrogels, we set to see if the TBA-based hydrogel can undergo further enzymatic degradation and transform into soluble hydrophilic triblock polymers (Figure 1). Following the rather slow enzymatic degradation of TBA at the micellar state, to expedite the enzymatic degradation, we used a 3-fold higher concentration of PLE in comparison to the previous conditions. In addition, bovine serum albumin (BSA) was added to both the samples containing the enzyme and the controls ones. We chose BSA because albumins are transport proteins, which are abundant in blood and known to interact nonspecifically with hydrophobic molecules and polymeric chains.^55,56^ As we have previously reported, this nonspecific interaction can shift the equilibrium toward the unimer state and hence expedite their enzymatic hydrolysis.^57^ Photos of the vials show the stability of the hydrogel in the presence of BSA and its full degradation into soluble hydrolyzed polymers in the presence of PLE, yielding a clear purple solution due to the presence of both Cy-3- and Cy-5-labeled polymers (Figure 2d).

To examine if the transition from micelles to hydrogel might cause a burst release of encapsulated cargo, we used Nile Red as model cargo and studied the formed micelles and hydrogels using fluorescence spectroscopy and confocal microscopy. The initial micellar solution showed the expected Nile Red emission at ∼640 nm (Figure 3a). Following the addition of PLE, a slow decrease in fluorescent emission is observed during the first 3 h, followed by a large drop at around 4 h, which can be explained by precipitation of the hydrogel as observed in the 5 h photo (Figure 3b). Eventually, after 24 h, the concentration of the dye in the solution drops almost to zero, indicative of the efficient encapsulation and retention of the cargo molecules during the mesophase transition. The confocal microscopy images (Figure 3c) show diffuse fluorescence of the entire micellar sample as the individual micelles are too small to be directly observed. This diffused emission shifts into localized hydrogel aggregates with strong emission due to the concentrating effect on the dyes upon the enzymatically induced gelation, demonstrating the potential of using such programmable formulations that can transform from micellar nanocarriers into drug depots.

**Figure 3:**
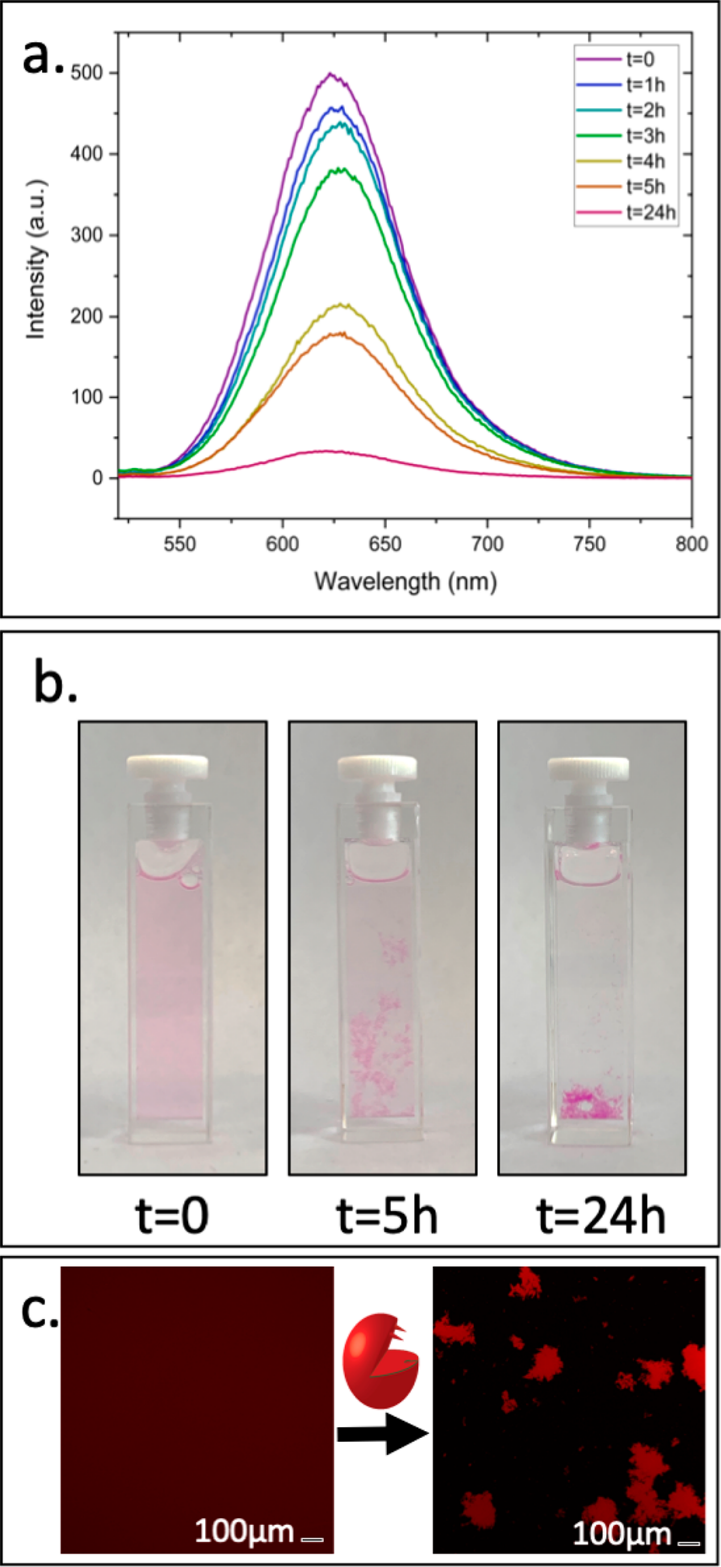
(a) Fluorescence spectra of micellar Nile Red solution over time. (b) Photos of the cuvettes at different time points. (c) Confocal images of the sample before and after PLE addition.

After confirming the transformation of the micelles into hydrogels, which can then slowly transform into soluble polymers, we wanted to test if we can program the time frame of the transition from micelles to hydrogels. To examine this, we increased the concentration of the DBA to prolong the time it takes for the enzyme to reach the critical DBA concentration and hence slowing down the overall transition to hydrogels. By doubling the concentration of DBA, we could achieve micelles of similar size (Figures S14b and S17b) that remained stable in the absence of the enzyme (Figures S16 and S17b) and transitioned into hydrogels after 10 h (Figures 4 and S26) when incubated with PLE—nearly twice the time it took for the 1:1 formulation. A comparison of the HPLC date for the two experiments (Figures 1e and 4b) shows that for both 1:1 and 2:1 formulations the critical gelation ratios had a nearly similar value of ∼0.3:1 DBA:TBA, at which the transition between the two mesophases occurs. These results show the ability to tune the time frame of the mesophase transitions by adjusting the composition of the formulation.

**Figure 4:**
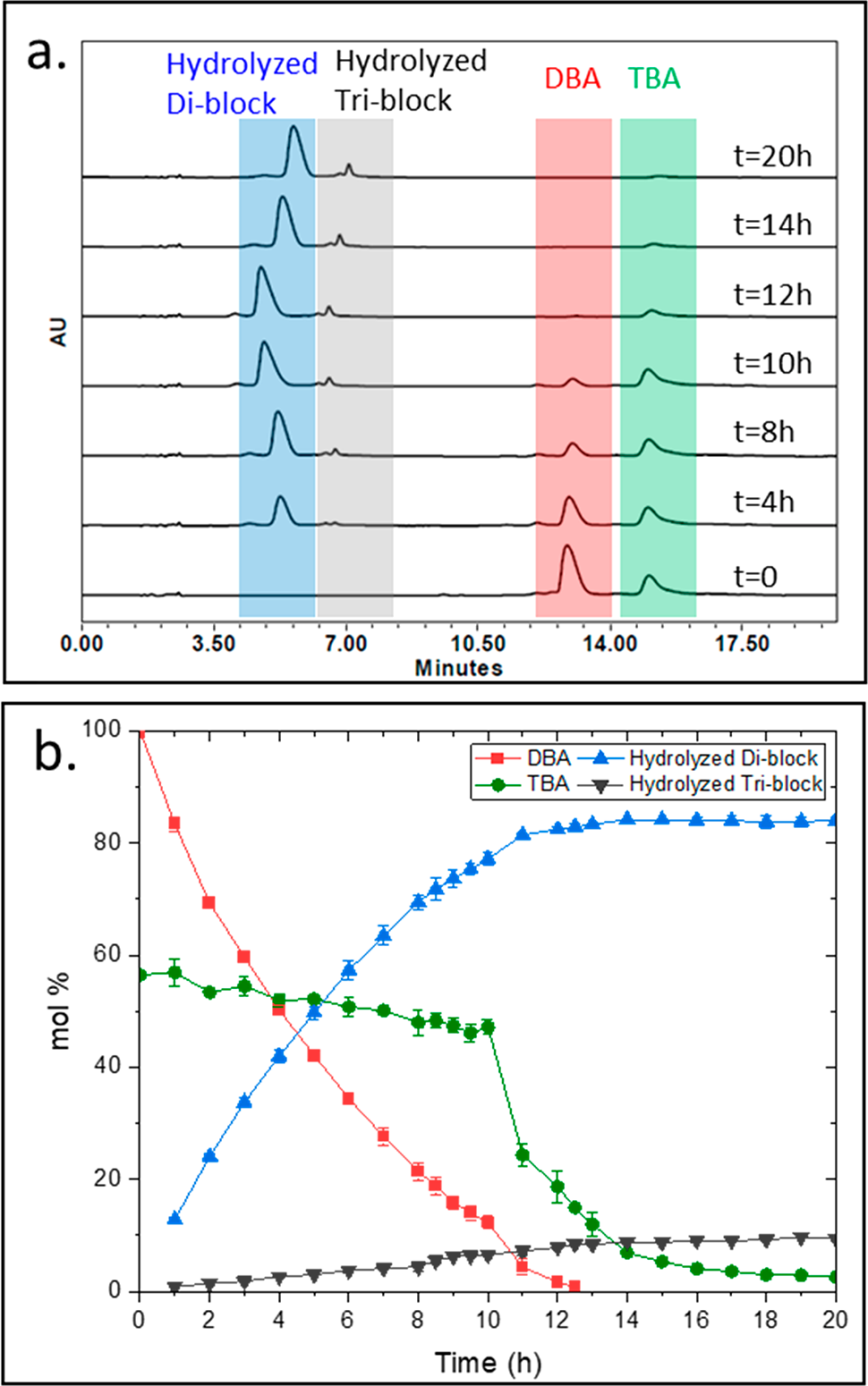
(a) HPLC data and (b) analysis of the enzymatic degradation of the amphiphiles (2:1 ratio).

In summary, we demonstrate the use of molecular architecture as a tool for programming sequential mesophase transitions. It is striking to see that despite having identical HLB and enzymatically cleavable groups, the different architectures and molecular weights significantly affected the reactivity of the two types of amphiphiles. Upon enzymatic activation, DBA got selectively degraded, and the DBA:TBA ratio decreased until the amount of DBA could no longer stabilize the micellar state and TBA aggregated into hydrogels. Upon further incubation with the enzyme, the formed TBA-based hydrogels underwent slow transition into soluble polymers. Importantly, we demonstrate that the composition of the formulation can be tuned to program the timing of the mesophase transitions. In addition, we show the potential formation of a hydrogel depot that can maintain the cargo that was encapsulated in the micelles. This proof of concept can be potentiality extended to design micellar nanocarriers that, upon encountering disease associated enzymes, will be able to transition into a hydrogel-based drug depot aimed at slow and sustained release of their encapsulated cargo, followed by their final degradation and clearance of the body after completing their task.

## Supporting Information Available

Synthetic procedures, amphiphiles and micelles characterization data, detailed experimental protocols and control experiments (PDF)
