# Supplementary material for: Architecture-Based Programming of Polymeric Micelles to Undergo Sequential Mesophase Transitions
Source: ACS Macro Lett. 2023 Jun 5;12(6):814–20. doi: 10.1021/acsmacrolett.3c00153 (PMC10286552; doi:10.1021/acsmacrolett.3c00153)
Supplement: Supplementary file 1 — mz3c00153_si_001.pdf [file mz3c00153_si_001.pdf]

Supporting Information

# Architecture-based programming of polymeric micelles to undergo sequential mesophase transitions

Parul Rathee,[‡a,b,c] Nicole Edelstein-Pardo,[‡a,b,c] Francesca Netti,[b,c,d] Lihi Adler-Abramovich,[b,c,d,e] Amit Sitt,[a,b,c,e] and Roey J. Amir[*,a,b,c,e]

[a]School of Chemistry, Faculty of Exact Sciences, Tel-Aviv University, Tel-Aviv 6997801, Israel

[b]The Center for Physics and Chemistry of Living Systems, Tel-Aviv University, Tel Aviv 6997801, Israel

[c]The Center for Nanoscience and Nanotechnology, Tel-Aviv University, Tel Aviv 6997801, Israel

[d]Department of Oral Biology, The Goldschleger School of Dental Medicine, Faculty of Medicine, Tel Aviv University, Tel Aviv 6997801, Israel

[e]ADAMA Center for Novel Delivery Systems in Crop Protection, Tel-Aviv University, Tel Aviv 6997801, Israel

## Table of Contents


1. Instrumentation ........ 3

2. Materials ........ 3
3. Synthesis ........ 4
   3.1 Preparation of di-block amphiphiles (DBA) ........ 4
   3.2 Preparation of tri-block amphiphiles (TBA) ........ 7
4. Characterization of DBA and TBA ........ 13
4.1 Gel permeation chromatography (GPC) ........ 13
4.2 Dynamic light scattering (DLS) ........ 15
4.3 Transmission electron microscopy (TEM) ........ 15
5. General Sample Preparation and Procedure of Measurement: ........ 17
   5.1 Enzymatic degradation experiments of mixed micellar formulations: ........ 17
   5.2 Enzymatic degradation experiments of non-mixed micellar formulations: ........ 18
   5.3 Characterization of the formed hydrogels: ........ 19
   5.4 Analysis of the composition of the formed hydrogels: ........ 22
   5.5 Fluorescence Measurements: ........ 23
   5.6 Hydrogel Degradation: ........ 23
   5.7 Nile Red Encapsulation: ........ 23
6. Gel formed from the tri-block copolymer in water ........ 24
7. Gel formed from the tri-block copolymer after the enzymatic degradation ........ 24

## 1. Instrumentation

**HPLC:** All measurements were recorded on a Waters Alliance e2695 separations module equipped with a Waters 2998 photodiode array detector. All solvents were purchased from Bio-Lab Chemicals and were used as received. All solvents are HPLC grade.

**$^{1}$H and $^{13}$C-NMR:** spectra were recorded on Bruker Avance III 400MHz/100MHz spectrometer. Chemical shifts are reported in ppm and referenced to the solvent. The molecular weights of the dendron-PEG-dendron tri-block copolymers were determined by comparison of the areas of the peaks corresponding to the PEG block (3.63 ppm) and the protons peaks of the dendrons.

**GPC:** All measurements were recorded on Viscotek GPC max by Malvern using refractive index detector and PEG standards (purchased from Sigma-Aldrich) were used for calibration. DMF (purchased from Sigma, HPLC grade) was used as the mobile phase. Columns (2 x PSS GRAM 1000Å) were used at a column temperature of 50°C.

**DLS:** All measurements were recorded on a Corduan Technology VASCO γ particle size analyzer.

**Fluorescence Spectra:** All spectra were recorded on an Agilent Technologies Cary Eclipse Fluorescence Spectrometer using quartz cuvettes.

**Confocal microscopy:** All images were taken in Olympus IX83 FLUOVIEW™ FV3000 inverted confocal microscope.

## 2. Materials

Poly(Ethylene Glycol) (10kDa), Poly(Ethylene Glycol) methyl ether (5kDa), allyl bromide (99%), 2,2-dimethoxy-2-phenylacetophenone (DMPA, 99%), Fmoc-Lys(Boc)-OH Novabiochem®, N,N'-Diisopropylcarbodiimide, Propargyl bromide, 80% solution in toluene, 4-nitrophenol (99.5%), 4-dimethylamino pyridine (DMAP), N,N'-dicyclohexylcarbodiimide (DCC, 99%), 2-mercaptoethanol, Indolium, 2,3,3-Trimenthylindolenine, Hexanoic acid, 6-Bromohexanoic acid, Acetic acid, Aniline, triethyl formate, Triethylsilane, and Sephadex® LH20 were purchased from Sigma-Aldrich. 3,5 dihydroxy benzoic acid and N-(3-(phenylimino)propenyl)aniline were purchased from Apollo Scientific Ltd. Anhydrous potassium carbonate, Sodium acetate and Trifluoroacetic acid (TFA) were purchased from Alfa Aesar. Potassium hydroxide, cystamine hydrochloride, Oxyma Pure Novabiochem® Triphenylmethyl chloride, Acetic anhydride, Diisopropylethylamine (DIPEA), Porcine liver esterase (PLE), and bovine serum albumin (BSA, Probumin®) were purchased from Merck. 2-(1H-Benzotriazole-1-yl)-1,1,3,3-tetramethyluronium hexafluorophosphate was purchased from Chem-Impex. Piperidine, Silica Gel 60Å 0.040-0.063mm, sodium hydroxide, and all solvents were purchased from Bio-Lab and were used as received. Deuterated solvents for NMR were purchased from Cambridge Isotope Laboratories (CIL).

## 3. Synthesis

### 3.1 Preparation of di-block amphiphiles (DBA)

mPEG$_{5k}$-Lys(Boc)-[dend-(yne)$_2$] **(1)** was synthesized as previously reported[1] and the spectroscopic characterization correlated well with this report.

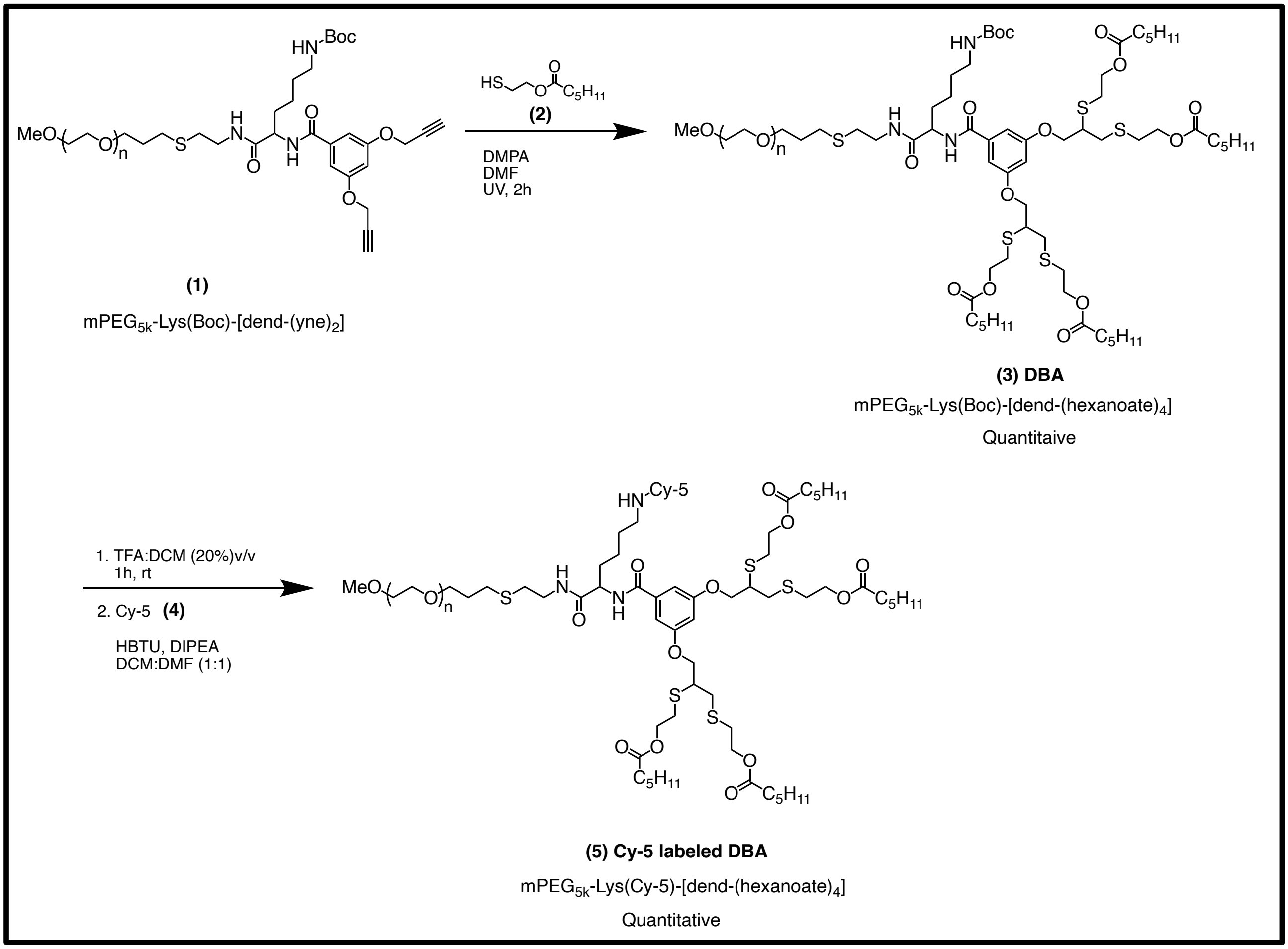


Figure S1: Synthetic scheme for the preparation of DBA and Cy-5 labelled DBA (mPEG$_{5k}$-Lys(Boc)-[dend-(hexanoate)$_4$] and mPEG$_{5k}$-Lys(Cy5)-[dend-(hexanoate)$_4$]), respectively.

**DBA (mPEG$_{5k}$-Lys(Boc)-[dend-(hexanoate)$_4$], compound 3):**

200 mg of compound **(1)**[1] (0.036 mmol) were dissolved in DMF (0.5 mL). Compound **(2)**[2] (254 mg, 1.44 mmol) and DMPA (3.7 mg, 0. 014 mmol) were added to the solution. The solution was purged with nitrogen for 15 minutes and then placed under UV light at 365 nm for 2 hours. The crude mixture was loaded on a MeOH-based LH20 SEC column. The fractions containing the product was unified, the MeOH was evaporated and further purification was done by re-dissolving the oily residue in DCM (1 mL) followed by precipitation by the dropwise addition of Ether (50 mL). The white precipitate was filtered, washed twice with Ether and dried under high vacuum. The product **(3)** was obtained as a white solid (quantitative yield).

$^1$H-NMR (400 MHz, $CDCl_3$): δ 7.02 (d, J = 7.5 Hz, 1H, Ar-**H**), 6.98 (d, J = 2.8 Hz, 2H, Ar-**H**), 6.68 (m, 1H, -$CH_2$-N**H**-CO-CH-), 6.60 (d, J = 2.6 Hz, 1H, -CH-N**H**-CO-C-), 4.68 (m, 1H, -N**H**-Boc), 4.54 (q, J = 7.3 Hz, 1H, -CO-C**H**-NH-), 4.33-4.12 (m, 12H, -Ar-O-C**H**$_2$- + C**H**$_2$-O-CO-$C_5$), 3.83-3.42 (m, PEG backbone), 3.36 (s, 3H, C**H**$_3$-O-PEG), 3.20 (q, J = 5.5 Hz, 2H, -C**H**-S-), 3.14-2.48 (m, 18H, -S-C**H**$_2$-$CH_2$-NH- + -C**H**$_2$-NH-Boc + -CH-C**H**$_2$-S- + -CH-$CH_2$-S-C**H**$_2$ + $CH_2$-C**H**$_2$-S-), 2.28 (t, J = 7.5 Hz, 8H, -O-CO-C**H**$_2$), 2.04-1.66 (m, 4H, -O-$CH_2$-C**H**$_2$-$CH_2$-S- + Boc-NH-$(CH_2)_3$C**H**$_2$-CH-), 1.54-1.38 (m, 13H, Boc-NH-$CH_2$-C**H**$_2$-C**H**$_2$-$CH_2$- + Boc), 1.35-1.15 (m, 24H, -O-CO-$CH_2$-C**H**$_2$ + -O-CO-$(CH_2)_2$-C**H**$_2$ + -O-CO-$(CH_2)_3$-C**H**$_2$), 0.86 (t, J=6.3 Hz 12H, -O-CO-$(CH_2)_4$-C**H**$_3$).

$^{13}$C-NMR (100 MHz, $CDCl_3$) δ 173.6, 171.4, 166.7, 159.4, 156.0, 136.0, 106.1, 104.6, 70.43, 63.31, 63.0, 59.0, 53.4, 45.4, 40.0, 38.5, 34.7, 34.1, 32.0, 31.4, 30.3, 29.5, 28.3, 28.1, 24.5, 22.7, 22.2, 13.8. GPC (DMF + 25 mM $NH_4Ac$): expected $M_n$=6.3 kDa, experimental $M_n$ = 6.3 kDa, $Đ_M$ = 1.08.

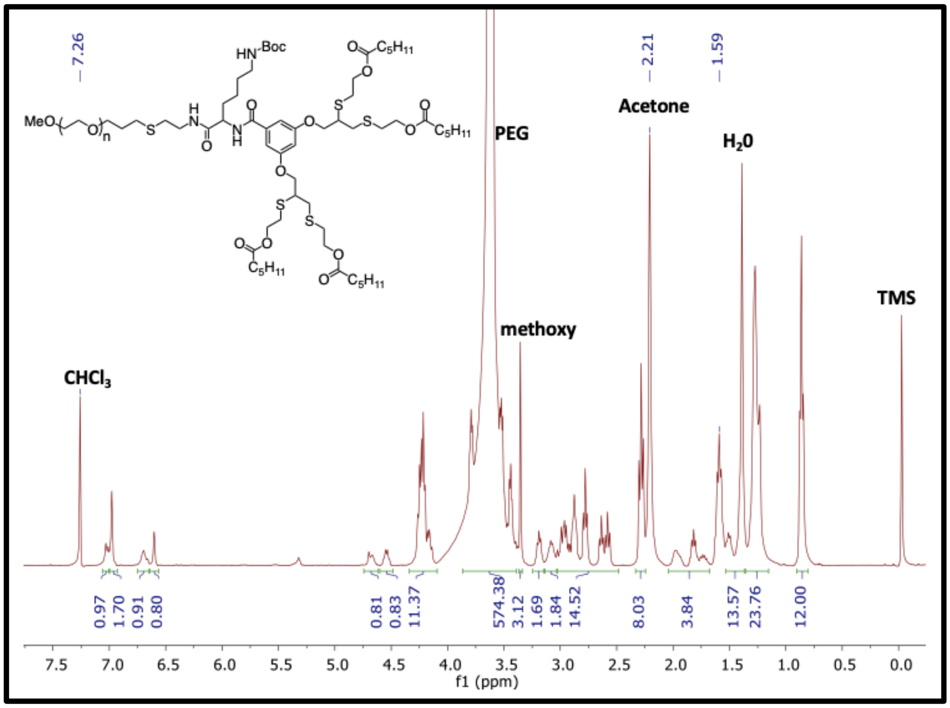


Figure S2: $^1$H-NMR spectrum of DBA (mPEG$_{5k}$-Lys(Boc)-[dend-(hexanoate)$_4$], compound 3) in $CDCl_3$.

Cy-5 fluorescent dye **(4)** was synthesized as previously reported[3] and the spectroscopic characterization correlated well with this report.

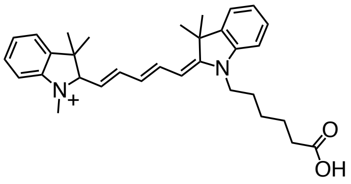

**(4)**

## Cy-5 labeled DBA (mPEG$_{5k}$-Lys(Cy-5)-[dend-(hexanoate)$_4$], compound 5):

100 mg (0.016 mmol) of compound **(3)** were dissolved in DCM (1 mL) and TFA (200 μL). The mixture was allowed to stir for 1 hour and the reaction was monitored via HPLC. Once the Boc deprotection was confirmed by HPLC, DIPEA was added to the reaction mixture until fumes from it stopped coming out. After that MeOH-based LH20 SEC column was done to get rid of TFA.

Cy-5 (29.3 mg, 0.048 mmol), HBTU (18.2 mg, 0.048 mmol) and DIPEA (31 mg, 0.239 mmol) were dissolved in a total volume of 1 mL DCM: DMF (1:1 v/v) in a 4 mL vial. The solution was stirred for 2 minutes and then it was added to the concentrated polymer hybrid purified by MeOH-based LH20 SEC column. The reaction mixture was allowed to stir for 2 hours and then loaded on a MeOH-based LH20 SEC column. The fractions containing the product was unified, the MeOH was evaporated and further dried under high vacuum. The product **(5)** was obtained as a dark blue solid (quantitative yield).

$^1$H-NMR (400 MHz, $CDCl_3$): see the following spectrum and assignments; $^{13}$C-NMR (100 MHz, $CDCl_3$): 173.5, 166.4, 159.3, 153.1, 152.1, 142.7, 140.9, 136.5, 128.8, 128.6, 126.2, 125.4, 124.8, 122.0, 111.1, 110.1, 106.4, 105.1, 104.7, 103.3, 70.4, 63.3, 63.0, 58.9, 54.5, 49.3, 45.5, 44.4, 39.2, 37.7, 35.9, 34.8, 34.0, 31.5, 31.2, 30.9, 30.2, 29.5, 28.3, 28.0, 26.9, 26.0, 25.0, 24.5, 22.7, 22.2, 13.8.

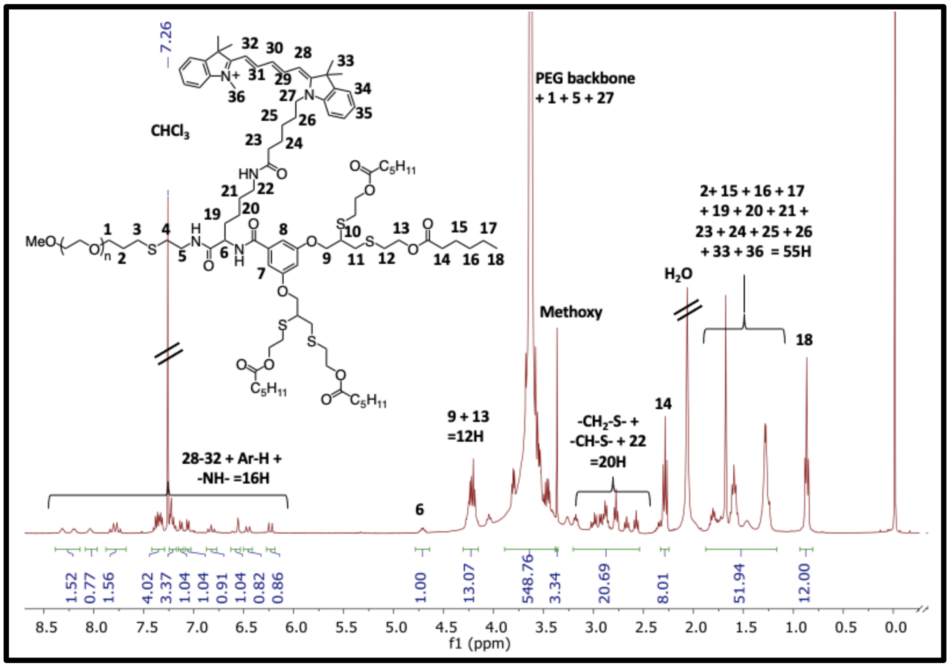


Figure S3: $^1$H-NMR spectrum of Cy-5 labeled DBA (mPEG$_{5k}$-Lys(Cy-5)-[dend-(hexanoate)$_4$], compound 5) in $CDCl_3$.

## 3.2 Preparation of tri-block amphiphiles (TBA)

bPEG$_{10k}$-bis-amine **(6)** was synthesized as previously reported[4] and the spectroscopic characterization correlated well with this report.

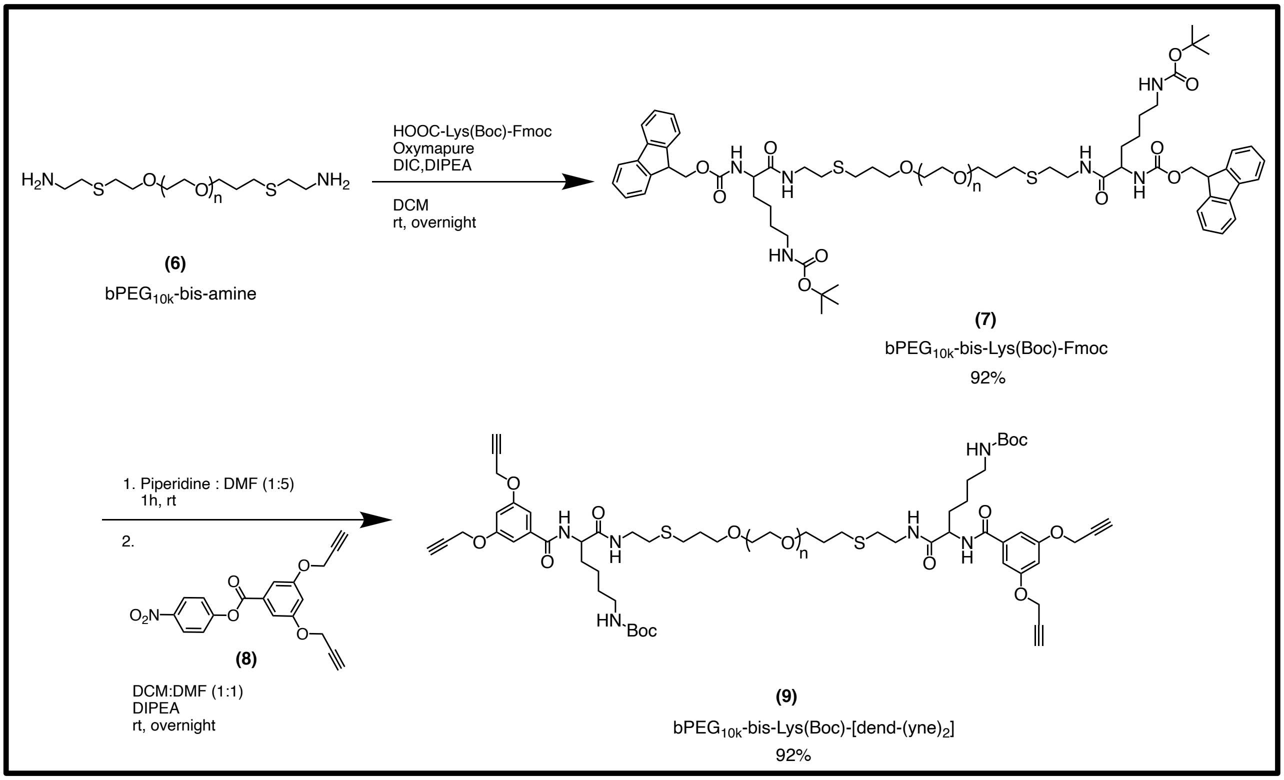


Figure S4: Synthetic scheme for the preparation of TBA, bPEG$_{10k}$-bis-Lys(Boc)-[dend-(yne)$_2$] (compound 9).

**bPEG$_{10k}$-bis-Lys(Boc)-Fmoc, compound 7:**

HOOC-Lys(Boc)-Fmoc (6 eq.) and DIC (6 eq.) were dissolved in DCM (2mL) followed by the addition of oxymapure (6 eq.) and DIPEA (6eq.). 600 mg (0.058 mmol) of bPEG$_{10k}$-bis-amine **(6)** was added to the above solution and the reaction mixture was stirred overnight at room temperature. The crude mixture was loaded on a MeOH-based LH20 SEC column. The fractions containing the product was unified, the MeOH was evaporated and further purification was done by re-dissolving the oily residue in DCM (1 mL) followed by precipitation with the dropwise addition of Ether (50 mL). The white precipitate was filtered, washed twice with Ether and dried under high vacuum. The product **(7)** was obtained as a white solid (92% yield).

$^1$H-NMR (400 MHz, $CDCl_3$): δ 7.69 (d, J = 7.5 Hz, 4H, Ar-**H**), 7.53 (d, J = 7.2 Hz, 4H, Ar-**H**), 7.33 (t, J = 7.4 Hz, 4H, Ar-**H**), 7.24 (d, J = 7.4 Hz, 4H, Ar-**H**), 6.60 (m, 2H, -CH$_2$-N**H**-CO-CH-), 5.59 (m, 2H, -N**H**-Fmoc), 4.71 (m, 2H, -N**H**-Boc), 4.35 (d, J = 6.2 Hz, 4H, Fmoc-C**H**$_2$-), 4.14 (t, J = 6.6 Hz, 2H, Fmoc-C**H**-CH$_2$-), 4.05 (m, 2H, -CO-C**H**-NH-), 3.57-3.31 (m, PEG backbone), 3.10-2.86 (m, 4H, Boc-NH-C**H**$_2$-), 2.64-2.49 (m, 8H, -C**H**$_2$-S-C**H**$_2$-), 1.88-1.49 (m, 8H, -O-CH$_2$-C**H**$_2$-CH$_2$-S- + Boc-NH-(CH$_2$)$_3$-C**H**$_2$-CH-), 1.36 (m, 26H, Boc-NH-CH$_2$-C**H**$_2$-C**H**$_2$-CH$_2$-CH- +

Boc); $^{13}$C-NMR (100 MHz, $CDCl_3$): δ 171.6, 156.2, 143.8, 141.3, 127.7, 127.1, 125.0, 120.0, 70.6, 70.1, 69.4, 66.9, 54.9, 47.2, 39.9, 38.5, 32.1, 31.5, 29.6, 28.4, 28.2, 22.5. GPC (DMF + 25 mM $NH_4Ac$): expected $M_n$= 11.1 kDa, experimental $M_n$ = 11.7 kDa, $Đ_M$ = 1.06.

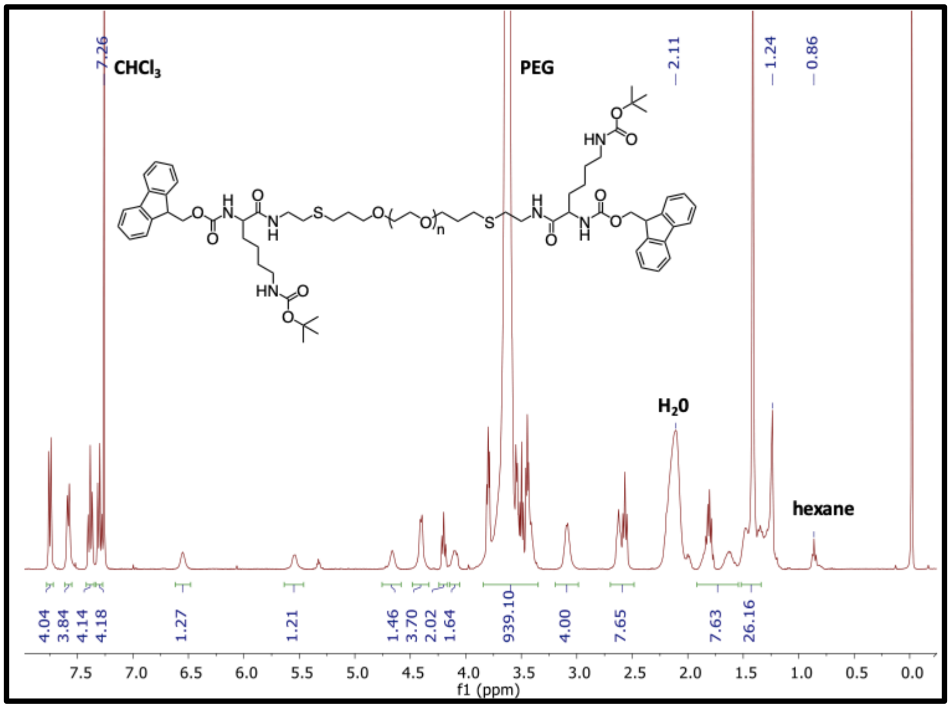


Figure S5: $^1$H-NMR spectrum of bPEG$_{10k}$-bis-Lys(Boc)-Fmoc (compound 7) in $CDCl_3$.

**bPEG$_{10k}$-bis-Lys(Boc)-[dend-(yne)$_2$], compound 9:**

400mg (0.036mmol) of bPEG$_{10k}$-bis-Lys(Boc)-Fmoc **(7)** were dissolved in 20% piperidine v/v in DMF (3 mL) and stirred for 1 hour. The deprotected product was precipitated by the dropwise addition of Ether (50 mL). The white precipitate was filtered and washed with Ether and dried under high vacuum. The deprotected product was obtained as a white solid. Compound **(8)**[5] (10 eq.) and HBTU (10 eq.) were dissolved in DCM: DMF 1:1 (1 mL) followed by the addition of DIPEA (20eq.). The solution was added to the deprotected bPEG$_{10k}$-bis-Lys(Boc)-$NH_2$ dissolved in DCM (1mL). The reaction was stirred overnight, and a negative Kaiser test confirmed complete coupling. The crude mixture was loaded on a MeOH-based LH20 SEC column. The fractions containing the product was unified, the MeOH was evaporated and further dried under high vacuum. The product **(9)** was obtained as a white solid (92% yield).

$^1$H-NMR (400 MHz, $CDCl_3$): δ 7.07 (d, J = 2.1 Hz, 4H, Ar-**H**), 6.97 (d, J = 7.4 Hz, 2H, -CH-N**H**-CO-Ar-), 6.85-6.70 (m, 4H, Ar-**H** + -$CH_2$-N**H**-CO-CH-), 4.72 (d, J = 2.2 Hz, 10H, -O-C**H**$_2$-C≡CH + -NH-Boc), 4.56 (q, J = 7.6 Hz, 2H, -CO-C**H**-NH-), 3.82-3.33 (m, PEG backbone), 3.18-3.02 (m, 4H, Boc-NH-C**H**$_2$-), 2.74-2.53 (m, 10H, -C**H**$_2$-S-C**H**$_2$- + -O-$CH_2$-C≡C**H**), 2.08-1.68 (m, 8H, -O-$CH_2$-C**H**$_2$-CH2-S- + Boc-NH-$(CH_2)_3$-C**H**$_2$-CH-), 1.58 -1.35 (m, 26H, Boc-NH-$CH_2$-C**H**$_2$-C**H**$_2$-$CH_2$-CH- + Boc); $^{13}$C-NMR (100 MHz, $CDCl_3$) δ 171.4, 166.6, 158.6, 156.0, 136.1, 106.8, 105.6,

81.9, 76.0, 70.4, 69.3, 56.1, 53.4, 41.8, 38.5, 32.1, 31.4, 29.6, 29.5, 28.3, 28.1, 22.6. GPC (DMF + 25 mM $NH_4Ac$): expected $M_n$= 11.1 kDa, experimental $M_n$ = 12.3 kDa, $Đ_M$ = 1.06.

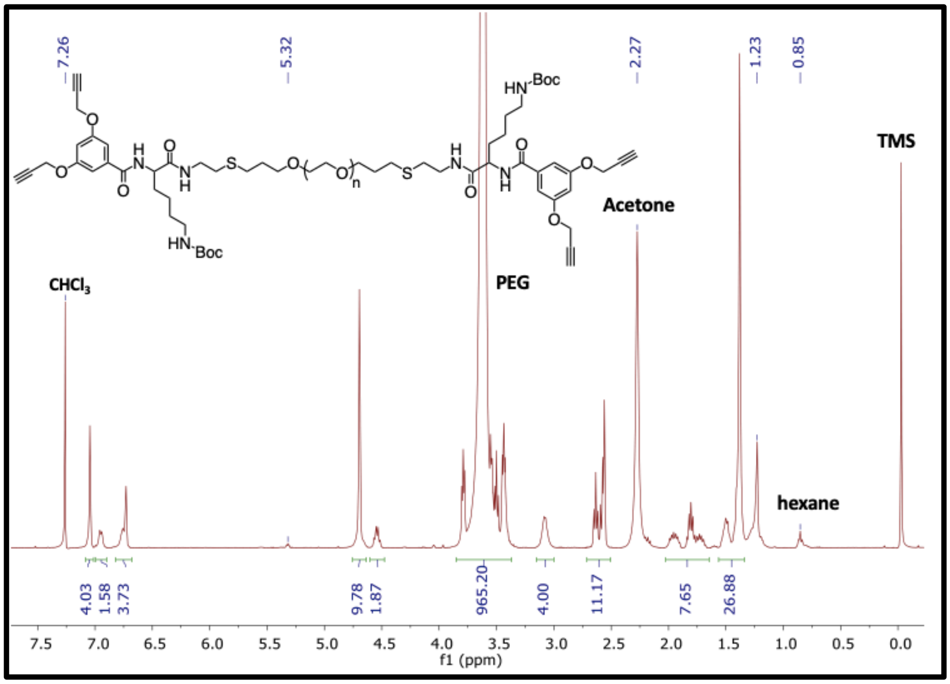


Figure S6: $^{1}$H-NMR spectrum of $bPEG_{10k}$-bis-Lys(Boc)-[dend-$(yne)_2$], compound 9 in $CDCl_3$.

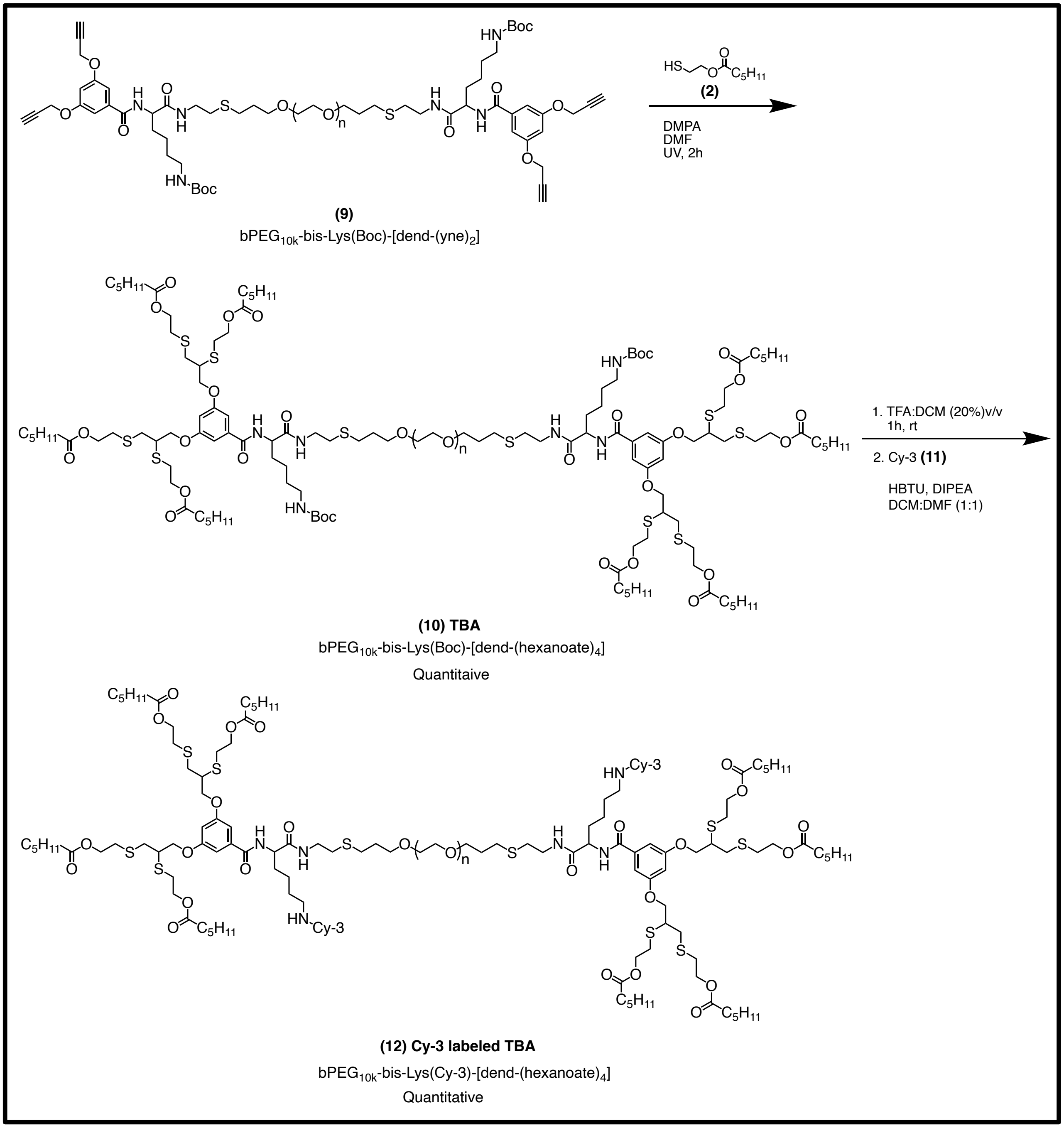


Figure S7: Synthetic scheme for the preparation of Cy-3 labeled TBA (bPEG$_{10k}$-bis-Lys(Cy-3)-[dend-(hexanoate)$_4$]).

## **TBA (bPEG$_{10k}$-bis-Lys(Boc)-[dend-(hexanoate)$_4$], compound 10):**

200 mg (0.036 mmol) of compound **(9)** were dissolved in DMF (0.5 mL). Compound **(2)**[2] (254 mg, 1.44 mmol) and DMPA (3.7 mg, 0.014 mmol) were added to the solution. The solution was purged with nitrogen for 15 minutes and then placed under UV light at 365 nm for 2 hours. The crude mixture was loaded on a MeOH-based LH20 SEC column. The fractions containing the

product was unified, the MeOH was evaporated and further purification was done by re-dissolving the oily residue in DCM (1 mL) followed by precipitation with the dropwise addition of Ether (50 mL). The white precipitate was filtered, washed twice with Ether and dried under high vacuum. The product **(10)** was obtained as a white solid (quantitative yield).

$^1$H-NMR (400 MHz, $CDCl_3$): δ 7.02 (d, J = 7.5 Hz, 2H, -CH-N**H**-CO-Ar-), 6.98 (d, J = 2.8 Hz, 4H, Ar-**H**), 6.68 (m, 2H, $-CH_2$-N**H**-CO-CH-), 6.60 (d, J = 2.6 Hz, 2H, Ar-**H**), 4.68 (m, 2H, -N**H-**Boc), 4.54 (q, J = 7.3 Hz, 2H, -CO-C**H**-NH-), 4.33-4.12 (m, 24H, -Ar-O-C$\mathbf{H_2}$- + C$\mathbf{H_2}$-O-CO-$C_5$), 3.83-3.42 (m, PEG backbone), 3.20 (q, J = 5.5 Hz, 4H, -C**H**-S-), 3.17-2.54 (m, 36H, Boc-NH-C$\mathbf{H_2}$- + -CH-C$\mathbf{H_2}$-S- + -CH-$CH_2$-S-C$\mathbf{H_2}$- + -NH-$CH_2$-C$\mathbf{H_2}$-S- + -$CH_2$-C$\mathbf{H_2}$-S-), 2.28 (t, J = 7.5 Hz, 16H, -O-CO-C$\mathbf{H_2}$), 2.04-1.66 (m, 8H, -O-$CH_2$-C$\mathbf{H_2}$-$CH_2$-S- + Boc-NH-$(CH_2)_3$C$\mathbf{H_2}$-CH-),1.54-1.38 (m, 26H, Boc-NH-$CH_2$-C$\mathbf{H_2}$-C$\mathbf{H_2}$-$CH_2$-CH- + Boc), 1.35-1.15 (m, 48H, -O-CO-$CH_2$-(C$\mathbf{H_2}$)$_3$-$CH_3$), 0.86 (t, J=6.3 Hz 24H, -O-CO-$(CH_2)_4$-C$\mathbf{H_3}$). $^{13}$C-NMR (100 MHz, $CDCl_3$) δ 173.5, 171.4, 166.6, 159.4, 156.0, 136.0, 106.1, 104.6, 70.4, 63.3, 63.0, 53.4, 45.4, 40.0, 38.5, 34.7, 34.1, 32.0, 31.4, 31.1, 30.3, 29.5, 28.3, 28.1, 24.5, 22.7, 22.2, 13.8. GPC (DMF + 25 mM $NH_4Ac$): expected $M_n$= 12.5 kDa, experimental $M_n$ = 13.7 kDa, $Đ_M$ = 1.08.

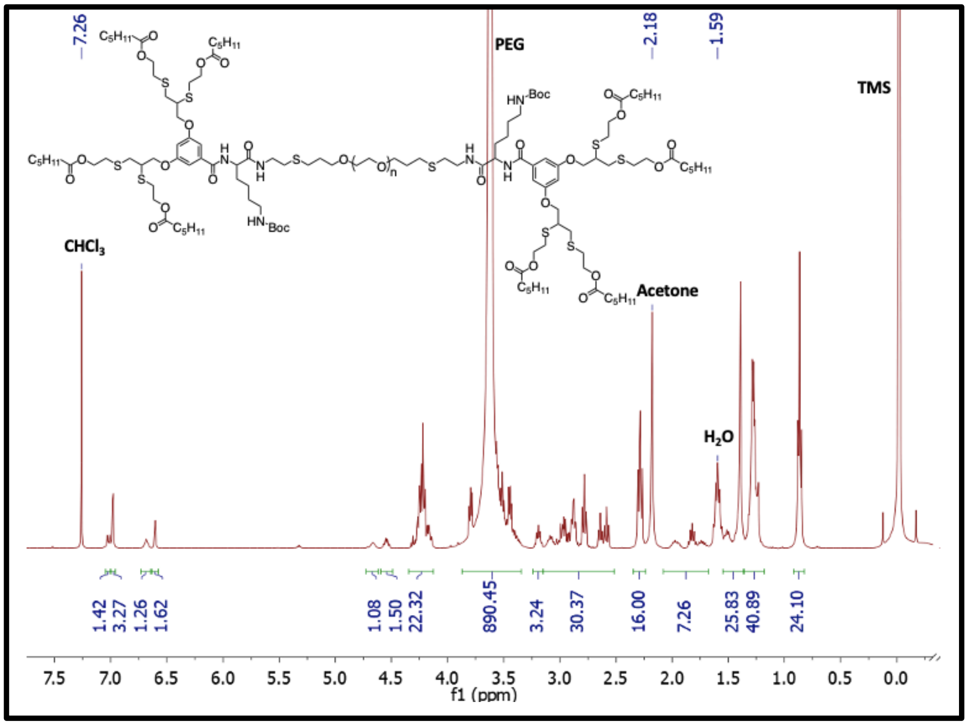


Figure S8: $^1$H-NMR spectrum of TBA (bPEG$_{10k}$-bis-Lys(Boc)-[dend-(hexanoate)$_4$], compound 10) in $CDCl_3$.

Cy-3 fluorescent dye **(11)** was synthesized as previously reported[3] and the spectroscopic characterization correlated well with this report.

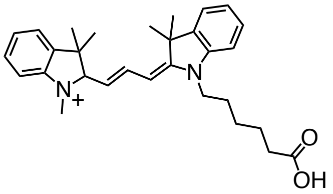


**(11)**

**Cy-3 labeled TBA (bPEG$_{10k}$-bis-Lys(Cy-3)-[dend-(hexanoate)$_4$], compound 12):**

100 mg (0.008 mmol) of compound **(10)** were dissolved in DCM (1 mL) and TFA (200 μL). The mixture was allowed to stir for 1 hour and the reaction was monitored via HPLC. Once the Boc deprotection was confirmed by HPLC, DIPEA was added to the reaction mixture until fumes from it stopped coming out. After that MeOH-based LH20 SEC column was done to get rid of TFA.

Cy-3 (29.3 mg, 0.048 mmol), HBTU (18.2 mg, 0.048 mmol) and DIPEA (31 mg, 0.239 mmol) were dissolved in a total volume of 1 mL DCM: DMF (1:1 v/v) in a 4mL vial. The solution was stirred for 2 minutes and then it was added to the concentrated polymer hybrid purified by MeOH-based LH20 SEC column. The reaction mixture was allowed to stir for 2 hours and then loaded on a MeOH-based LH20 SEC column. The fractions containing the product were unified, and the MeOH was evaporated and further dried under high vacuum. The Cy-3 labeled TBA product **(12)** was obtained as a dark blue solid (quantitative yield).

$^{1}$H-NMR (400 MHz, $CDCl_3$): see following spectrum and assignments; $^{13}$C-NMR (100 MHz, $CDCl_3$): 173.5, 166.4, 159.3, 150.7, 141.9, 136.5, 129.0, 128.8, 125.5, 125.4, 122.0, 111.2, 111.0, 106.4, 105.1, 104.5, 104.0, 70.4, 63.4, 63.1, 58.8, 54.1, 49.0, 45.6, 44.6, 39.2, 38.7, 38.0, 37.8, 36.0, 34.8, 34.0, 31.5, 31.2, 31.0, 30.2, 29.5, 28.2, 28.1, 26.7, 26.0, 25.0, 24.5, 22.7, 22.2, 13.8.

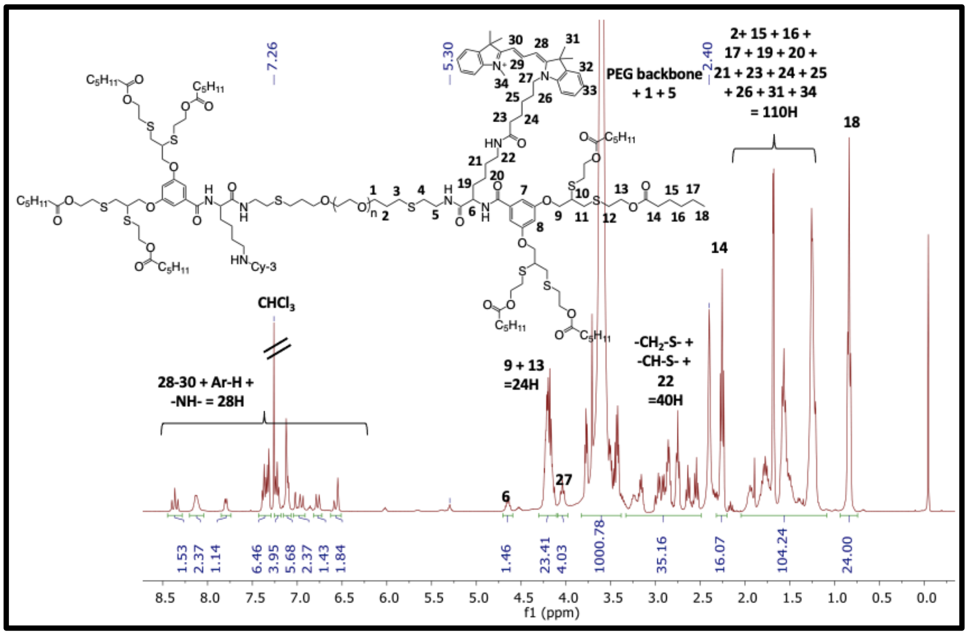


Figure S9: $^1$H-NMR spectrum of Cy-3 labeled TBA (bPEG$_{10k}$-bis-Lys(Cy-3)-[dend-(hexanoate)$_4$], compound 12) in $CDCl_3$.

## 4. Characterization of DBA and TBA

### 4.1 Gel permeation chromatography (GPC)

Instrument method: Instrument: Malvern Viscotek GPCmax

Columns: 2xPSS GRAM 1000 Å

Columns temperature: 50°C

Flow rate: 0.5 mL/min

Injection time: 60 min

Injection volume: 50 µL from a 10 mg/mL sample

Diluent + mobile phase: DMF + 25 mM $NH_4Ac$

Needle wash: DMF

Detector: Viscotek VE3580 RI detector

Sample preparation: The amphiphiles were directly dissolved in the diluent to give a final concentration of 10 mg/mL and filtered with 0.22 µm PTFE syringe filter.

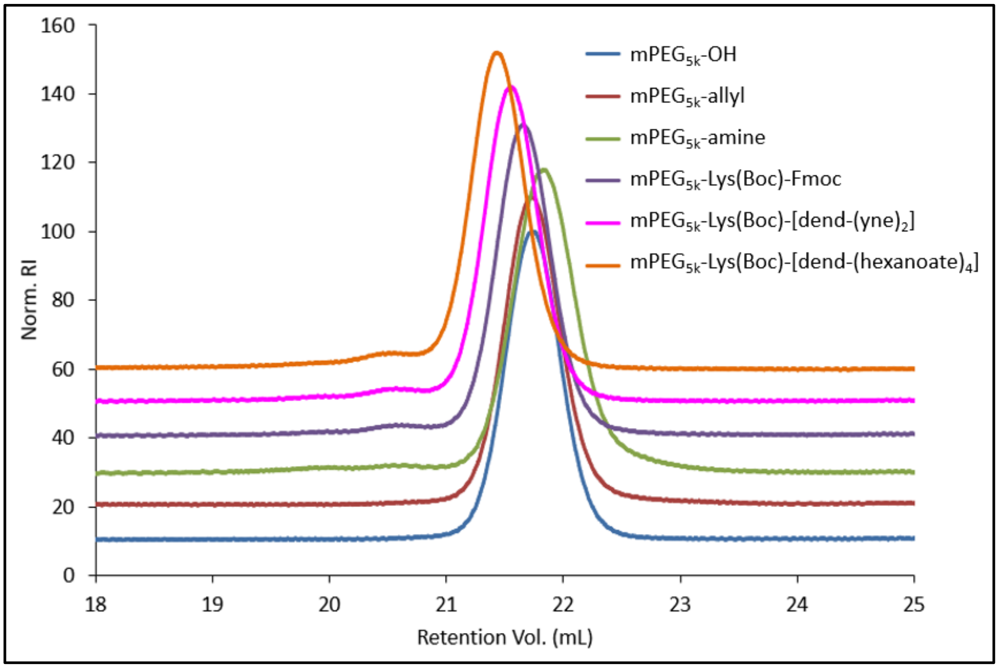


Figure S10: GPC traces overlay of commercial 5kDa methoxy PEG (blue), $mPEG_{5k}$-allyl (red), $mPEG_{5k}$-amine (green), $mPEG_{5k}$-Lys(Boc)-Fmoc (purple), $mPEG_{5k}$-Lys(Boc)-[dend-$(yne)_2$] (sky blue) and DBA, $mPEG_{5k}$-Lys(Boc)-[dend-$(hexanoate)_4$] (orange).

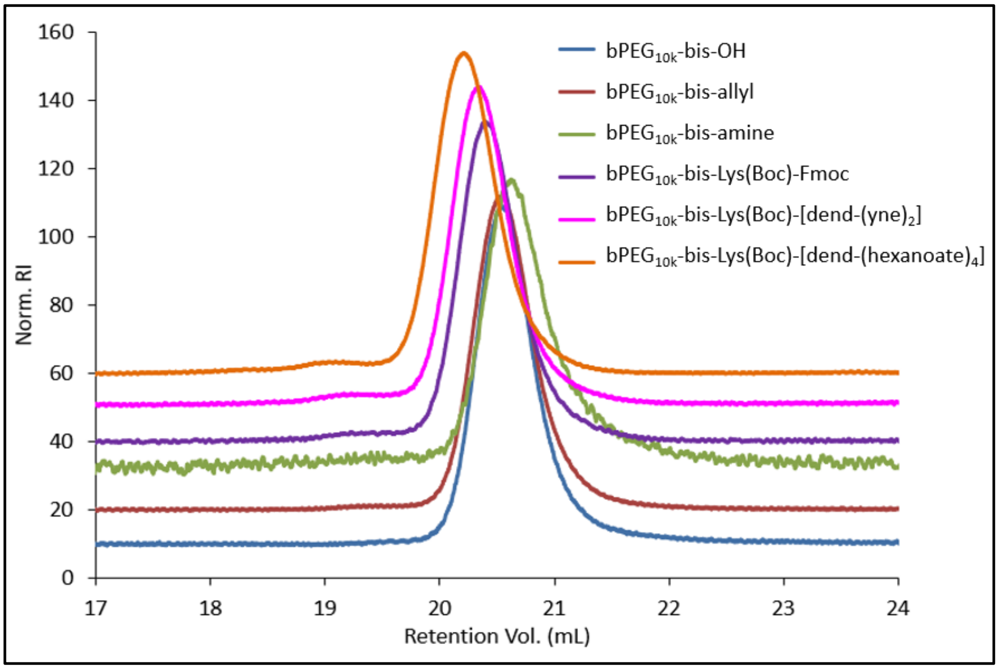


Figure S11: GPC traces overlay of commercial 10kDa bPEG (blue), $bPEG_{10k}$-bis-allyl (red), $bPEG_{10k}$-bis-amine (green), $bPEG_{10k}$-bis-Lys(Boc)-Fmoc (purple), $bPEG_{10k}$-bis-Lys(Boc)-[dend-$(yne)_2$] (pink) and TBA, $bPEG_{10k}$-bis-Lys(Boc)-[dend-$(hexanoate)_4$] (orange).

## 4.2 Dynamic light scattering (DLS)

**General sample preparation:**

A micellar solution (1:1 DBA: TBA) was prepared by mixing 5mg of each DBA (compound 3) and TBA (compound 10) in 1mL PBS giving a total polymers concentration of 10 mg/mL. Similarly, for the 2:1 DBA: TBA, 10mg of DBA (compound **3**) and 5mg of TBA (compound **10)** in 1 mL PBS give a total concentration of 15mg/mL. Vials were vortexed until full solubility was obtained, and then the solutions were sonicated for 15 minutes and filtered through a 0.22 µm nylon syringe filter. Measurements were performed at t=0 before the addition of the PLE enzyme.

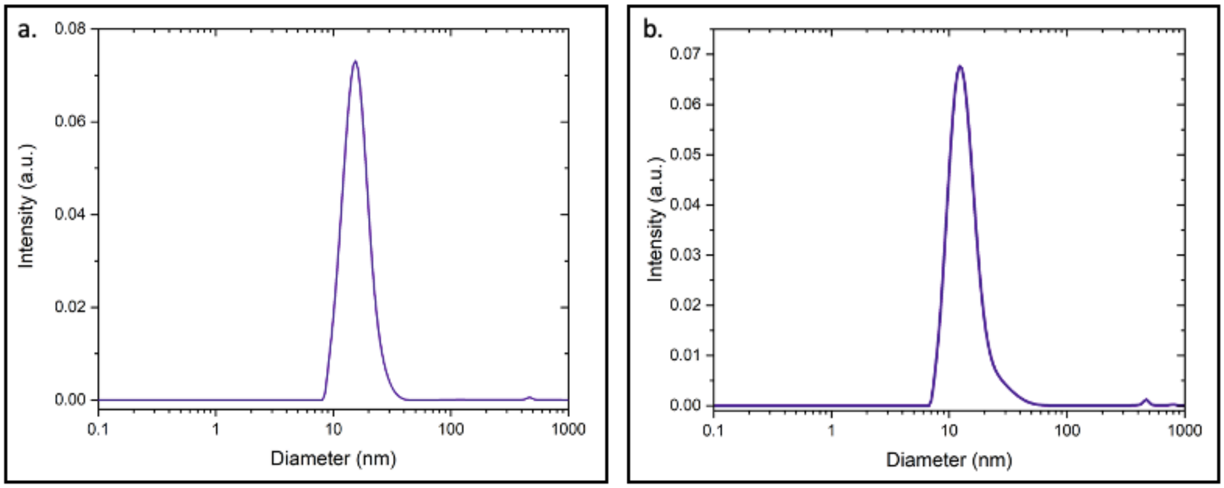


Figure S12: DLS results for co-assembled DBA (compound 3) and TBA (compound 10) in micellar form (t=0). (a) 1:1 ratio and (b) 2:1 ratio.

## 4.3 Transmission electron microscopy (TEM)

**General sample preparation:**

A micellar solution of DBA was prepared by mixing 10mg of DBA (compound 3) in 1mL PBS to give a polymer concentration of 10mg/mL. This solution was then furhter diluted in PBS to prepare another solution of concentration 5mg/mL.

A micellar solution (1:1 DBA: TBA) was prepared by mixing 5mg of each DBA (compound 3) and TBA (compound 10) in 1mL PBS giving a total polymers concentration of 10 mg/mL. Similarly, for the 2:1 DBA: TBA, 10mg of DBA (compound **3**) and 5mg of TBA (compound **10**) in 1 mL PBS give a total concentration of 15mg/mL. Vials were vortexed until full solubility was obtained, and then the solutions were sonicated for 15 minutes and filtered through a 0.22 µm nylon syringe filter. Measurements were performed at t=0 before the addition of PLE enzyme.

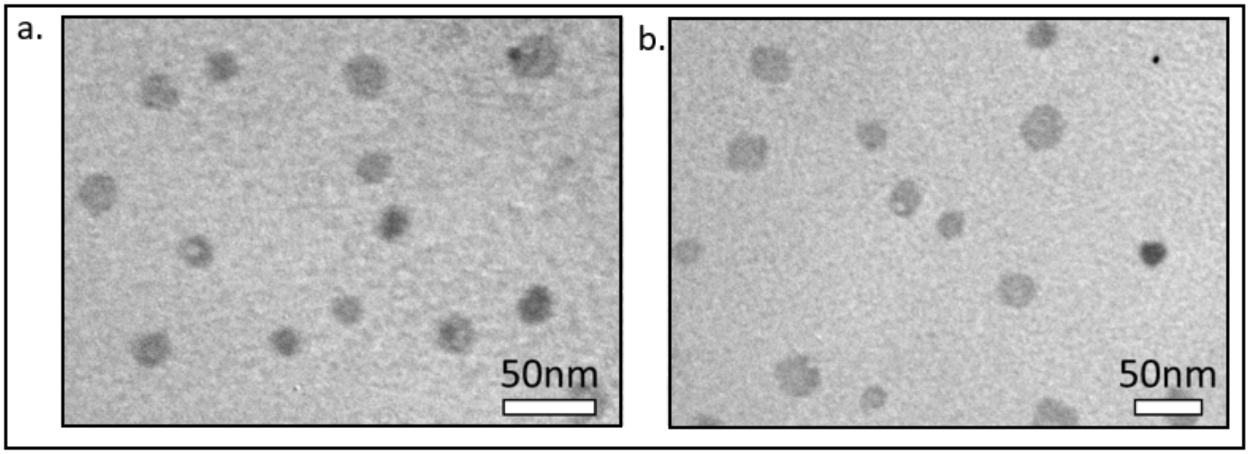


Figure S13: TEM images for micelles formed from DBA at different concentrations. (a) 5mg/mL and (b) 10mg/mL.

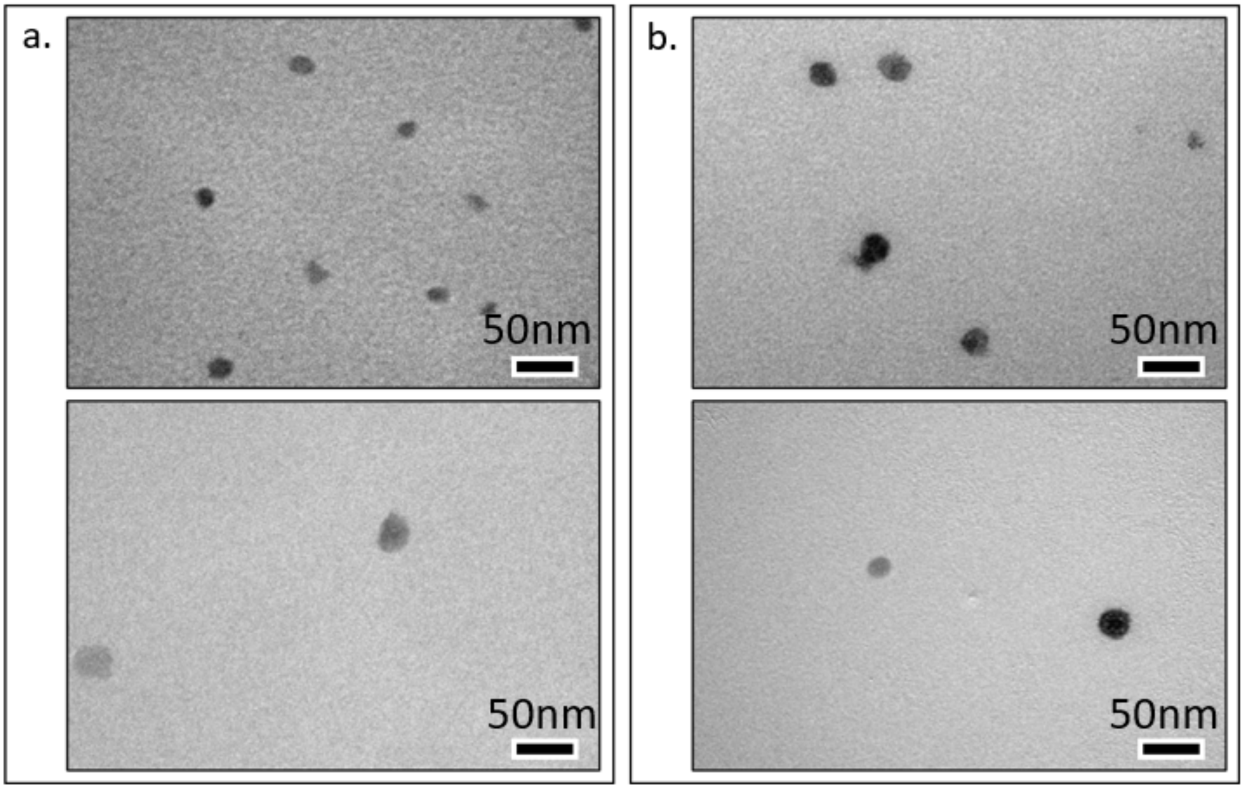


Figure S14: TEM images for co-assembled micelles (t=0). (a) DBA (compound 3) and TBA (compound 10) in 1:1 ratio and (b) in 2:1 ratio.

# 5. General Sample Preparation and Procedure of Measurement:

## 5.1 Enzymatic degradation experiments of mixed micellar formulations:

A micellar solution (1:1 DBA: TBA) was prepared by mixing 5mg of each DBA (compound **3**) and TBA (compound **10**) in 1mL PBS giving a total polymers concentration of 10 mg/mL. Similarly, for the 2:1 DBA: TBA, 10mg of DBA (compound **3**) and 5mg of TBA (compound **10**) in 1 mL PBS give a total concentration of 15mg/mL. Vials were vortexed until full solubility was obtained and then placed in an ultrasonic bath for 15 minutes. PLE was added to yield a final concentration of 0.36 μM and degradation was followed at 37°C by monitoring the area under the peak of the parent amphiphile and hydrolyzed polymer by HPLC at 297 nm. Each experiment was conducted thrice; the reported values in each time point are the mean value, and the standard deviation is the error (results shown in main text, Figures 1 and 4).

To monitor the thermodynamic stability of the mixed micelles obtained from mixing DBA and TBA in 1:1 and 2:1, control experiments were done without adding enzyme. The degradation was followed at 37°C by monitoring the area under the peak of the parent amphiphile by HPLC at 297 nm. Each experiment was conducted thrice; the reported values in each time point are the mean value, and the standard deviation is the error.

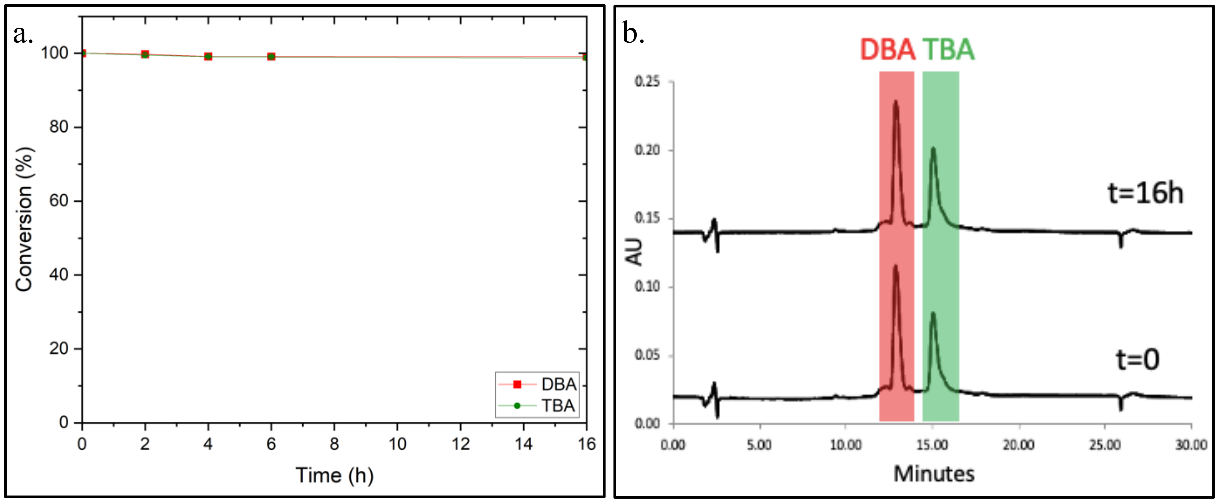


Figure S15: DBA (compound 3) and TBA (compound 10) in 1:1 ratio in the absence of enzyme (a) HPLC monitoring and (b) HPLC overlay.

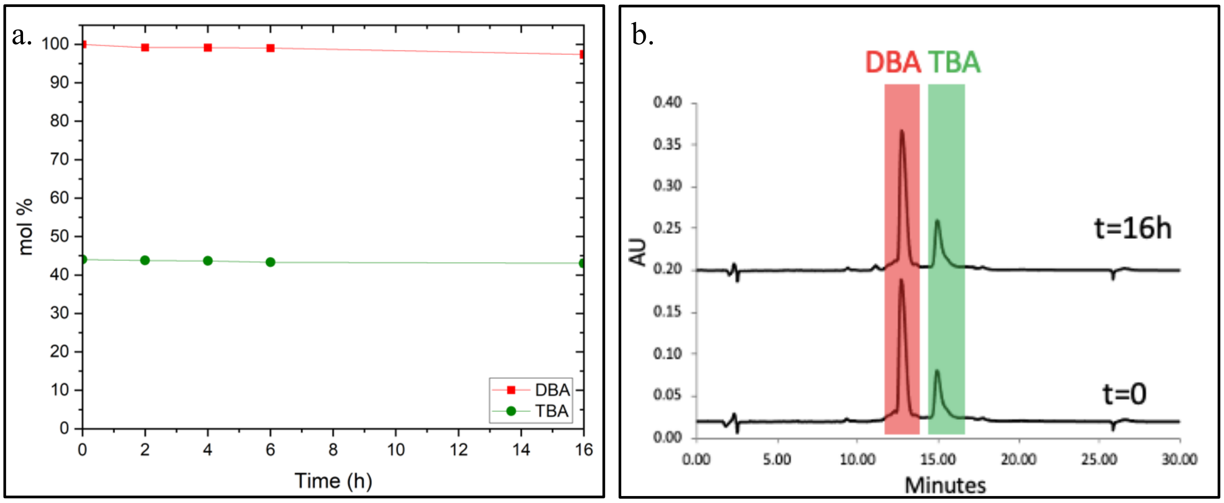


Figure S16: DBA (compound 3) and TBA (compound 10) in 2:1 ratio in the absence of enzyme (a) HPLC monitoring and (b) HPLC overlay.

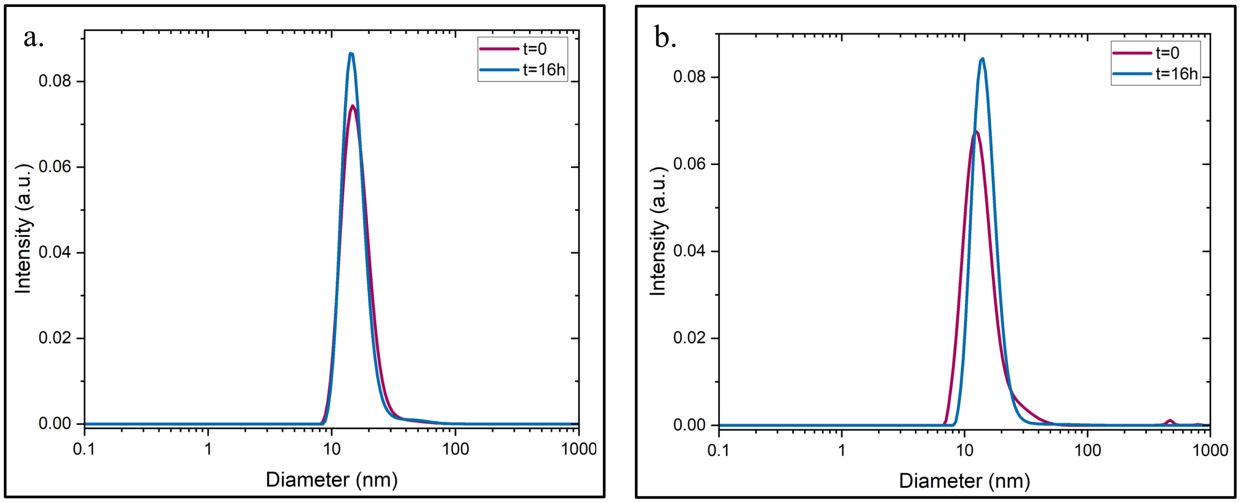


Figure S17: DLS results for co-assembled DBA (compound 3) and TBA (compound 10) at t=0 and t=16h in the absence of enzyme. (a) 1:1 ratio and (b) 2:1 ratio.

## 5.2 Enzymatic degradation experiments of non-mixed micellar formulations:

5mg/mL solution of DBA was prepared in PBS. Vials were vortexed until full solubility was obtained and then placed in an ultrasonic bath for 15 minutes. PLE was added to yield a final concentration of 0.36 μM and degradation was followed at 37°C by monitoring the area under the peak of the parent amphiphile and hydrolyzed polymer by HPLC at 297 nm. Each experiment was

conducted thrice; the reported values in each time point are the mean value, and the standard deviation is the error.

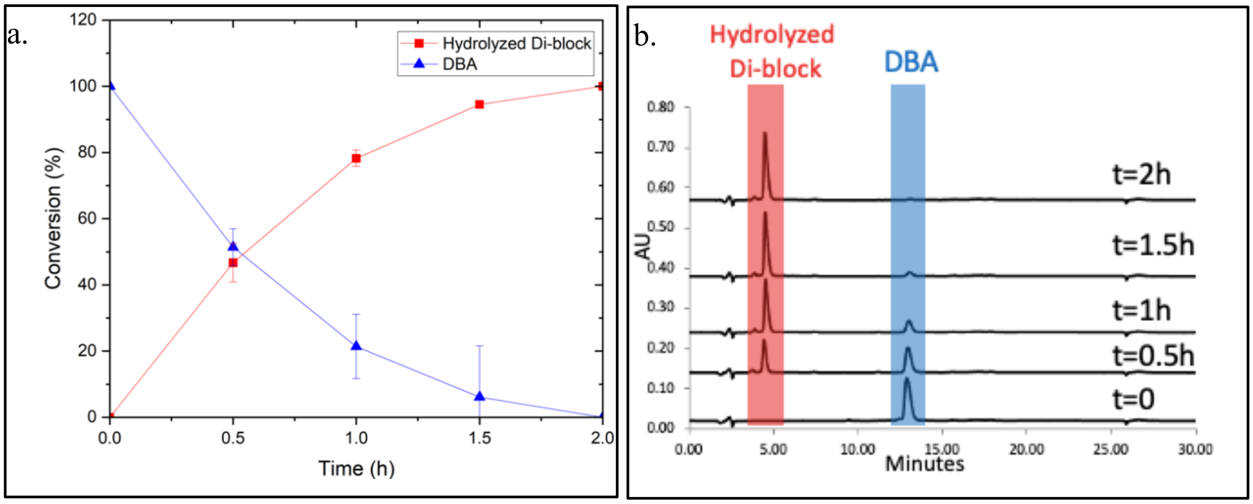


Figure S18: DBA (compound 3), 5mg/mL upon incubation with PLE (a) HPLC monitoring and (b) HPLC overlay.

For DLS, the solution was filtered through a 0.22 µm nylon syringe filter and measurements were done before and after micellar disassembly.

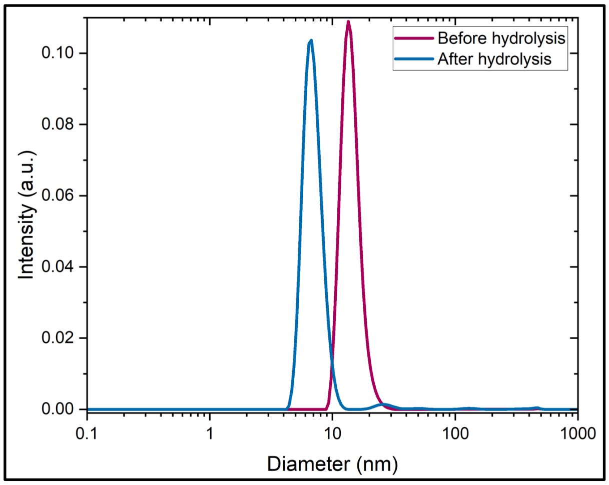


Figure S19: DLS results for DBA (compound 3) at t=0 and t=2h upon incubation with PLE.

## 5.3 Characterization of the formed hydrogels:

### 5.3.1 **Rheology measurements:**

Rheological measurements were performed using a controlled- stress rheometer (AR-G2, TA instruments, USA). An 8 mm diameter flat-plate geometry with a rough surface was used for the

study. The viscous elastic region was determined by strain sweep from 0.01 to 100% strain at 1Hz frequency at 25°C, with a gap size of 0.9 mm.

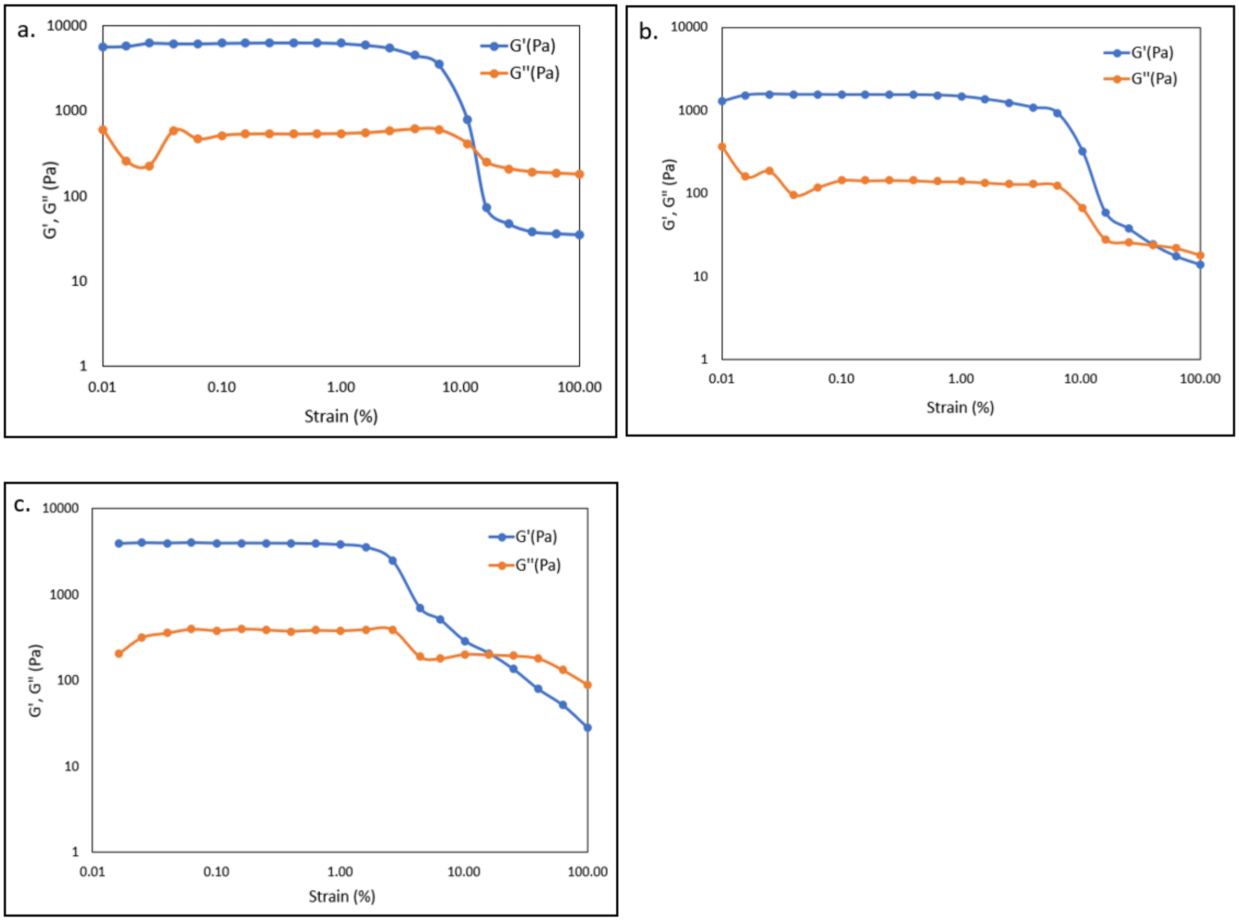


Figure S20: Amplitude sweep tests of the hydrogels obtained from (a) 1:1 DBA (compound 3, 5 mg/mL) and TBA (compound 10, 5 mg/mL), (b) in 2:1 DBA (compound 3, 10 mg/mL) and TBA (compound 10, 5 mg/mL), and (c) only TBA (5 mg/mL) at a constant frequency of 1Hz.

### 5.3.2 HRSEM measurements:

All images were taken using a Zeiss Gemini 300 high resolution scanning electron microscope in high vacuum, WD~5mm, 3kV.

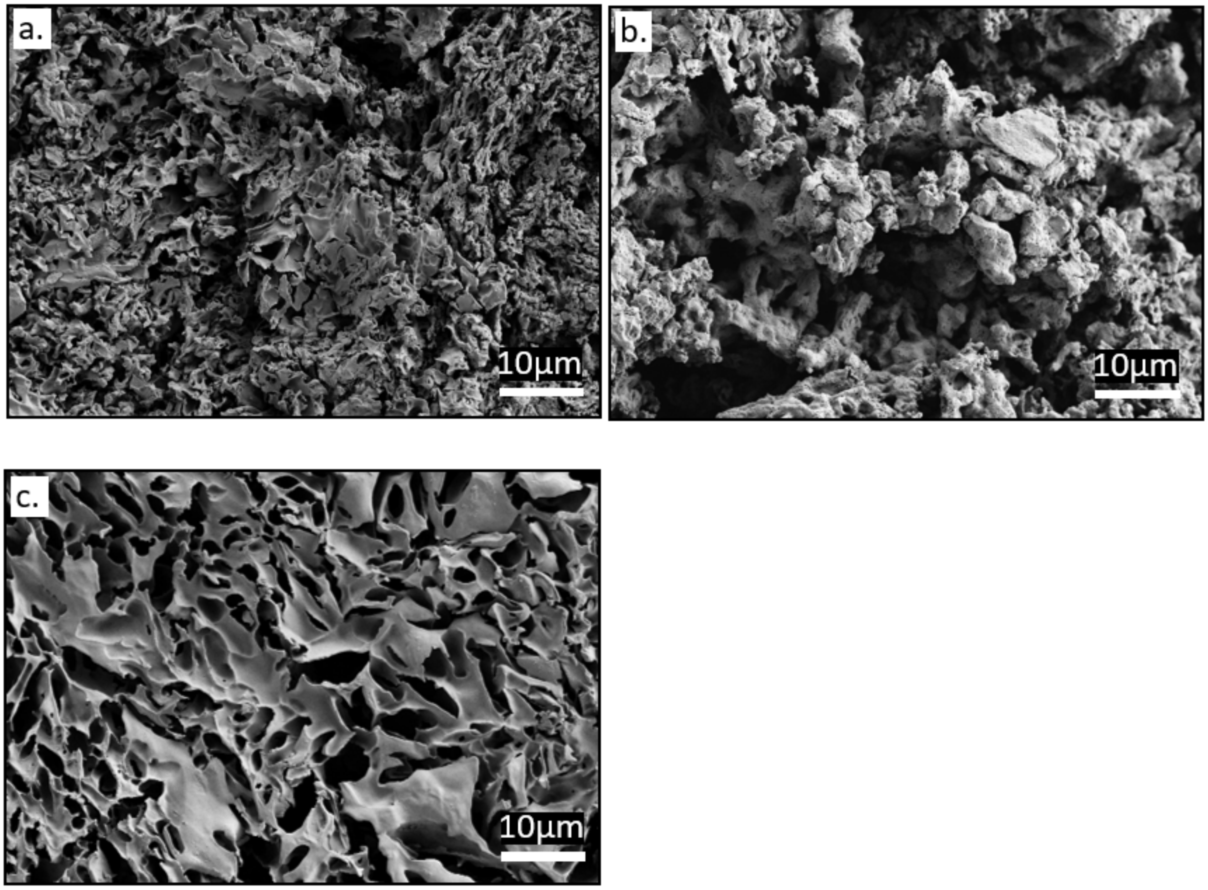


Figure S21: HRSEM images of the lyophilized hydrogels obtained from (a) DBA (compound 3) and TBA (compound 10) in 1:1 ratio, (b) in 2:1 ratio and (c) only TBA.

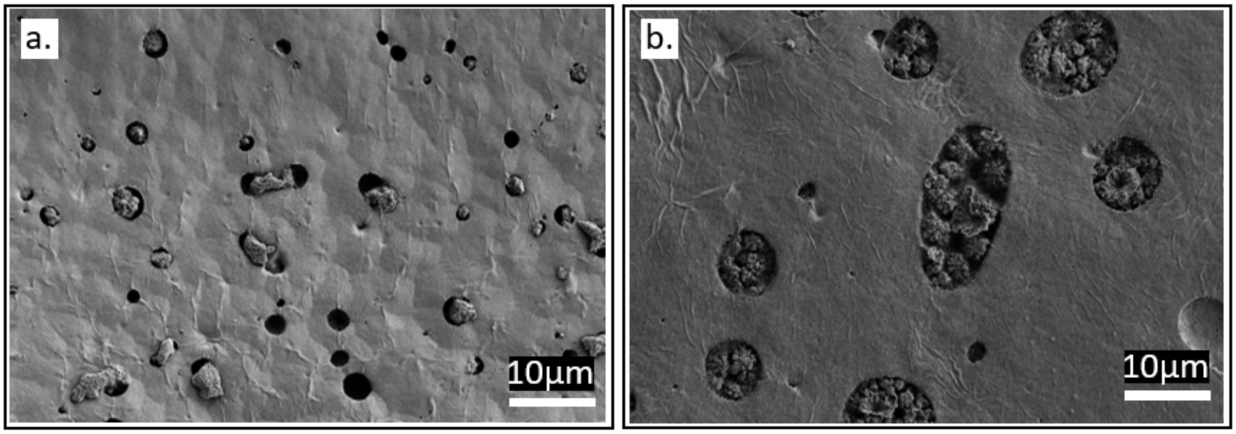

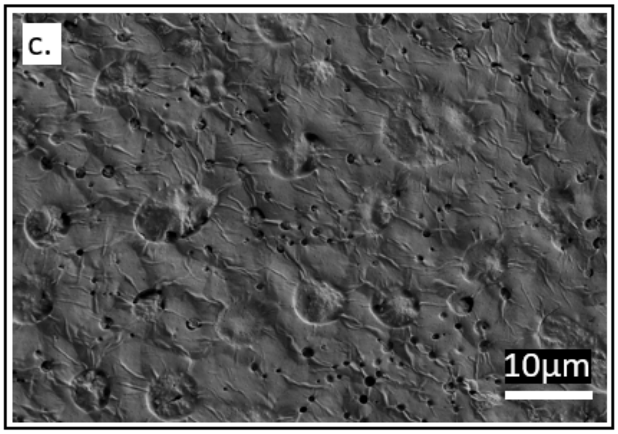


Figure S22: HRSEM images of the spontaneously dried hydrogels obtained from (a) DBA (compound 3) and TBA (compound 10) in 1:1 ratio, (b) in 2:1 ratio and (c) only TBA.

### 5.4 Analysis of the composition of the formed hydrogels:

The solution above the hydrogel was removed and the remaining hydrogel was washed 3 times with PBS and then dissolved in acetonitrile. The HPLC analysis shows the presence of 11% partly hydrolyzed amphiphiles, 12% DBA and 77% TBA.

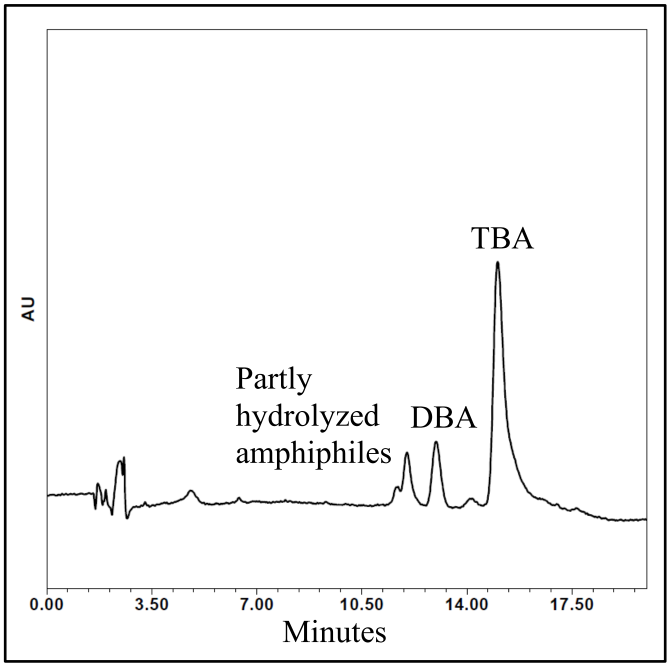


Figure S23: HPLC result after adding acetonitrile to the gel formed after the degradation of the di-block amphiphiles.

**5.5 Fluorescence Measurements:**

A micellar solution (1:1 DBA: TBA) was prepared by mixing 4.5 mg of each DBA (compound **3**) and TBA (compound **10**) and 0.5 mg of each compound: Cy-5 labeled DBA (**5**) and Cy-3 labeled TBA **(12)** in 1 mL PBS giving a total polymers concentration of 10mg/mL. The solution was vortexed until full solubility was obtained and then placed in an ultrasonic bath for 15 minutes. PLE was added to yield a final concentration of 0.36 µM and fluorescence was measured at 37°C by exciting at 512 nm (Cy-3 excitation), shown in main text, Figure 2a.

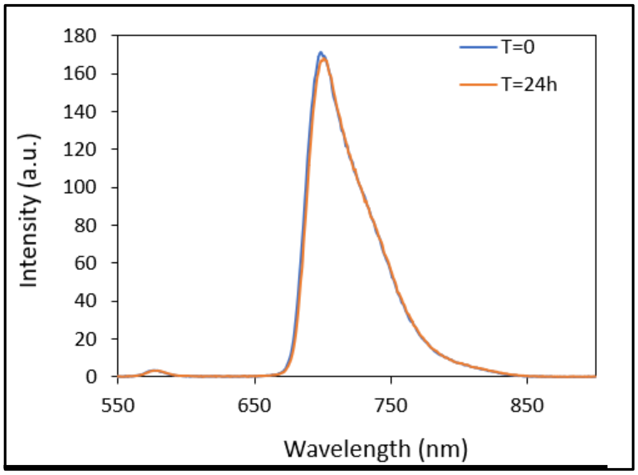


Figure S24: Fluorescence spectrum of micelles containing both dyes at t=0 and t=24h in the absence of PLE.

**5.6 Hydrogel Degradation:**

To study the stability of hydrogel formed from the enzymatic degradation experiment (Section 5.1) we added BSA and an excess of an enzyme. The solution above the hydrogel was removed and the remaining hydrogel was washed 3 times with PBS. Two parallel experiments were conducted, first, 500µL of 3.5mg/mL of BSA in PBS was added and second, 500µL of 3.5mg/mL of BSA along with 1µM of PLE in PBS was added.

**5.7 Nile Red Encapsulation:**

A micellar solution (1:1 DBA: TBA) was prepared by mixing 5 mg of each DBA (compound **3**) and TBA (compound **10**) and Nile red in 1 mL PBS giving a total polymeric concentration of 10 mg/mL and 10 µM of Nile red. The sample was vortexed until full solubility was obtained and then placed in an ultrasonic bath for 15 minutes. PLE was added to yield a final concentration of 0.36 µM and degradation was followed at 37°C by measuring the fluorescence by exciting at 500 nm.

## 6. Gel formed from the tri-block copolymer in water

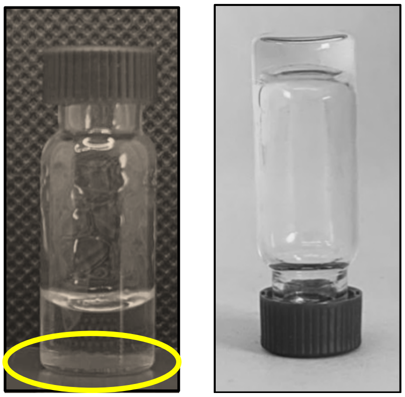

Figure S25: Gel formed from TBA (compound 10) in water with a concentration of 5mg/0.5mL using the thin-film hydration in the left vial, and 5mg/0.25mL using solvent exchange method in the right vial (ethanol was used as a solvent).

## 7. Gel formed from the tri-block copolymer after the enzymatic degradation

In the first experiment DBA (compound **3**, 5mg/mL) and TBA (compound **10**, 5mg/mL) were mixed in a 1:1 ratio with a total final concentration of 10 mg/mL, while in the second experiment DBA (compound **3**, 10mg/mL) and TBA (compound **10**, 5mg/mL) were mixed in a 2:1 ratio with a total final concentration of 15 mg/mL, the enzyme (PLE) concentration was 0.36 μM.

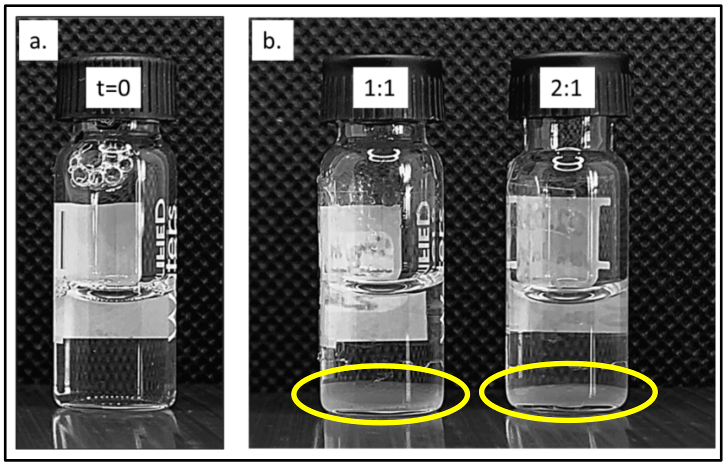


Figure S26: (a) Solution formed from DBA (compound 3) and TBA (compound 10) at t=0 before the addition of PLE. (b) Gel formed (at the bottom of the vials) in the two ratios tested.

References:


1. Buzhor. M; Harnoy, A. J.; Tirosh. E; Barak. A; Schwartz. T; and Amir. R. J. Supramolecular Translation of Enzymatically Triggered Disassembly of Micelles into Tunable Fluorescent Responses. *Chem. Eur. J.* **2015**, *21*, 15633–15638.

2. Wulf. V; Slor. Gadi; Rathee. Parul ; Amir. R. J. ; and Bisker. G. Dendron-Polymer Hybrids as Tailorable Responsive Coronae of Single-Walled Carbon Nanotubes. *ACS Nano.* **2021**, *15*, 20539−20549.

3. Jung. M. E. and Kim. W. J. Practical syntheses of dyes for difference gel electrophoresis. *Bioorganic and Medicinal Chemistry*. **2006**, *15*, 92-97.

4. Edelstein-Pardo N.; Molco. M; Rathee. P; Koren. G; Tevet. S; Sharabani. S. Z; Beck. R; Amir. R. J. ; and Sitt. A. Anisotropic Microparticles through Periodic Auto-Fragmentation of Amphiphilic Triblock Copolymers Microfibers. *Chem. Mater.* **2022**, *34*, 6367-6377.

5. Harnoy, A. J.; Rosenbaum, I.; Tirosh, E. Ebenstein, Y. Shaharabani, R.; Beck, R; and Amir. R. J. Enzyme-Responsive Amphiphilic PEG-Dendron Hybrids and Their Assembly into Smart Micellar Nanocarriers. *Chem. Soc.* **2014**, *136*, 7531-7534.
